# The Effect of Doping on the Electrical and Dielectric Properties of Hydroxyapatite for Medical Applications: From Powders to Thin Films

**DOI:** 10.3390/ma17030640

**Published:** 2024-01-28

**Authors:** Liviu Duta, Valentina Grumezescu

**Affiliations:** National Institute for Lasers, Plasma and Radiation Physics, 409 Atomistilor St., 077125 Magurele, Romania

**Keywords:** biomaterials, hydroxyapatite powders, pellets, and thin films, doping, electrical and dielectric properties, biomedical applications

## Abstract

Recently, the favorable electrical properties of biomaterials have been acknowledged as crucial for various medical applications, including both bone healing and growth processes. This review will specifically concentrate on calcium phosphate (CaP)-based bioceramics, with a notable emphasis on hydroxyapatite (HA), among the diverse range of synthetic biomaterials. HA is currently the subject of extensive research in the medical field, particularly in dentistry and orthopedics. The existing literature encompasses numerous studies exploring the physical–chemical, mechanical, and biological properties of HA-based materials produced in various forms (i.e., powders, pellets, and/or thin films) using various physical and chemical vapor deposition techniques. In comparison, there is a relative scarcity of research on the electrical and dielectric properties of HA, which have been demonstrated to be essential for understanding dipole polarization and surface charge. It is noteworthy that these electrical and dielectric properties also offer valuable insights into the structure and functioning of biological tissues and cells. In this respect, electrical impedance studies on living tissues have been performed to assess the condition of cell membranes and estimate cell shape and size. The need to fill the gap and correlate the physical–chemical, mechanical, and biological characteristics with the electrical and dielectric properties could represent a step forward in providing new avenues for the development of the next-generation of high-performance HA-doped biomaterials for future top medical applications. Therefore, this review focuses on the electrical and dielectric properties of HA-based biomaterials, covering a range from powders and pellets to thin films, with a particular emphasis on the impact of the various dopants used. Therefore, it will be revealed that each dopant possesses unique properties capable of enhancing the overall characteristics of the produced structures. Considering that the electrical and dielectric properties of HA-based biomaterials have not been extensively explored thus far, the aim of this review is to compile and thoroughly discuss the latest research findings in the field, with special attention given to biomedical applications.

## 1. Introduction

From a medical perspective, restoring the normal functionality of injured bone tissue is a significant task. Thus, dentistry and orthopedics still face significant challenges associated with both bone healing and growth processes. To enhance the healing process of fractured bones and ensure successful integration and functioning within the human body, biomaterials designed for applications in bone tissue engineering must exhibit specific key properties. These characteristics include, but are not limited to, mechanical strength, bio-inertness, bio-functionality, biocompatibility, lack of toxicity, and absence of inflammatory reactions at the implantation site. These enhanced attributes, combined with a detailed focus on the electrical and dielectric properties of biomaterials, have been demonstrated to be critical for designing bone implant materials for contemporary medical applications. They provide essential information about the local environment, such as stability and porosity [1]. A prime example of such a biomaterial is hydroxyapatite (HA), a remarkable bioceramic belonging to the calcium phosphate family, known for its high dielectric constant [2]. Scientists have harnessed the potential of HA for medical use (including bone tissue engineering) due to its osteogenic properties, ability to form strong bonds with bone tissues, resemblance to the native apatite found in the human body, and natural occurrence in bones, teeth, cartilage, eggshells, corals, and exoskeletal tissues [3,4]. Beyond their applications in environmental pollution remediation [5], energy storage [6,7], and sensors [8], HA-based materials are commonly utilized as fillers and coatings for the fabrication of orthopedic and dental implants [9].

The presence of a dielectric material in the composition of bones and the correlation between electrical and dielectric characteristics with bone growth, regeneration, repair, and bonding have been demonstrated [7,10,11,12,13,14]. Consequently, there exists a strong interest related to the development of electrically active HA-based nanoceramics for hard tissue applications to provide an improved biological response, considering that these materials have been shown to be biocompatible, and to boost the bone growth [15,16] and fracture healing characteristics [17,18,19,20]. Although cells possess a negative charge, they are surrounded by counter ions capable of moving toward the cell surface when subjected to an applied electrical field, leading to the formation of an induced dipole moment [21]. This phenomenon is associated with the development of polarization in the HA system. The recognized significance of this induced polarization extends to the regeneration of bone tissues, as demonstrated in both in vitro and in vivo [22] tests. Studies demonstrating the partial regeneration of lost limbs in rats after applying an electric signal stimulation underscored the significance of biomaterials’ electrical characteristics [11]. It was shown that the electric fields have been utilized for treating osteonecrosis and osteoarthritis and stimulating bone healing processes [23,24]. In this context, surfaces with a negative charge have been linked to enhanced bone growth when compared with positively charged surfaces [25,26]. Given the fact that the percentage of HA in natural bones is known to be approximately 70% and that bone mass is directly proportional to the dielectric properties of the HA system [21], the study of the electrical and dielectric characteristics of both HA and bone is, therefore, imperative for the successful fabrication of bio-implants.

Nevertheless, pure HA is still recognized to exhibit deficiencies concerning both its (i) mechanical properties (brittleness and low mechanical strength and fracture toughness), which are crucial for load-bearing applications, and (ii) bioactivity, which is essential for enhancing bone regeneration and antibacterial activity. Another drawback to the use of HA in bone tissue engineering lies in the occurrence of infections caused by pathogenic microorganisms, in which, unfortunately, the use of antibiotics does not assure adequate protection against resistant bacterial strains. Therefore, to address these limitations and enhance the electrical, dielectric, morpho-structural, and mechanical properties of HA-based materials, the strategic doping of HA structure with various trace metal and nonmetal ions, as well as clusters, or by polymer blending (HA/polymer composites) has been explored as a viable solution [27,28]. These approaches could allow for the finetuning of material properties, thereby supporting sustained bone regeneration while also mitigating the risk of implant failure. HA has also been found to possess weak piezoelectric properties, which are significant for bone regeneration [29]. It should be noted that both the piezoelectric [15,30] and dielectric [31] characteristics of bone are directly influenced by the applied frequencies. Moreover, the electrical properties of human bone indicate that the bone’s dielectric constant depends on both its water content and the electric field’s applied frequencies. It is worth mentioning that the value of the dielectric constant of dry bone is comparable to the value corresponding with sintered HA [32]. Hence, through precise control of the density and dopants, it is possible to tailor the dielectric constant of the synthesized HA to closely resemble that of natural bones.

Taking all these aspects into consideration, the aim of the current work is to comprehensively review the reported results on the electrical and dielectric properties (i.e., impedance, AC conductivity, dielectric constant, dielectric loss, tangent loss, and permittivity) of HA-based materials, ranging from powders and pellets to thin films, with a particular focus on the impact of various dopants/blends used. Therefore, it will be revealed that each dopant/blend possesses unique and superior properties (to pristine HA), capable of enhancing the overall characteristics of the produced structures. Considering that the electrical and dielectric properties of HA-based biomaterials have not been extensively explored thus far, a compilation and thorough discussion on the latest research findings in the field, with special attention given to biomedical applications, will be delivered. To a certain extent, this review can be regarded as an attempt to bridge the current gap between a detailed correlation of the physical–chemical, mechanical, and biological characteristics and the electrical and dielectric properties to offer insights into the potential development of the next generation of HA-doped/blended biomaterials.

## 2. Literature Survey

To review the latest reports on the electrical and dielectric properties of HA, a digital search was conducted using Scopus, Web of Science, and Google Scholar. The search followed specific inclusion criteria: (i) articles written in English, (ii) studies focusing on the electrical and dielectric properties of HA-based materials, (iii) investigations involving both simple and doped/blended HA-based materials, (iv) HA-based materials reported in the form of powders, nanoparticles, pellets, and thin films, (v) studies with relevance to biomedical applications, and (vi) a publication period between 2017 and 2023. It is important to mention that this specific timeframe (i.e., 2017–2023) was chosen to offer readers a comprehensive continuation of the review study previously published by A. Das and D. Pamu in 2019 [33]. Following the review process, a total of 26 articles met these criteria and were further assessed in detail. A selection of the results reported in these articles was included and thoroughly discussed in the current review.

## 3. Cation- and Anion-Substituted Hydroxyapatite

It is important to mention that the introduction of ion substitution and the doping of transition elements into the HA matrix have opened new avenues for the utilization of HA-based materials in the realm of medical and pharmaceutical sciences [34,35,36].

In the subsequent sections, an overview of the biological performances of various agents used to dope/blend HA, on which we will focus our attention in this study, will be introduced.

### 3.1. Cerium

Cerium (Ce), an *f*-block rare earth element, has been shown to enhance bone’s metabolism and form a biomimetic HA layer after immersion for 2–3 weeks in simulated body fluids (SBF). The as-obtained HA layer displayed excellent corrosion resistance, while the ion release test indicated no signs of leakage of any harmful ions into the physiological system [34]. It is noteworthy that the Ce^3+^ cation (1.14 Å) shares a similar size with the Ca^2+^ ion (1.12 Å) and has a tendency to replace it in the HA structure. Moreover, Ce limits dental cavities by promoting precipitation and reducing the demineralization of bone. In this respect, Ce-doped HA coatings onto Ti implants have demonstrated strong antimicrobial effects against a plethora of bacteria and have been recognized for their regenerative characteristics [34,37,38,39,40]. CeO_2_ NPs, whether in their bare form or stabilized with surfactants, were demonstrated to have the capability to penetrate cells. Thus, they may potentially interfere with cellular components, leading to damage in cell division and, ultimately, inhibiting the growth of the organism [41].

### 3.2. Gallium

Gallium (Ga), a versatile element extensively utilized in nanotechnology [42,43], has also been recognized for its significant biological effects, including stress relief, the modulation of skeletal metabolism, and the inhibition of bone resorption, as well as its potential in treating osteoporosis and cancer-related problems [44,45]. It is noteworthy that Ga-doped HA ceramics have been demonstrated to play an important role in osteosynthesis, enhancing the bone growth process [46,47,48], and mimicking in vivo bone mineralization [49]. Moreover, Ga has been found to adsorb onto the crystal surface from metastable calcium phosphate (CaP) solutions, thereby inhibiting the growth rate of HA [50]. Through the adjustment of the composition of the amorphous/Ga phosphate surface layer and the overall Ga content, the release of Ga^3+^ ions within the bioactive range has also been demonstrated [46]. Given the fact that Ga-67 radioactive isotope has been shown to possess a strong bone affinity, the fabrication of Ga-doped HA ceramics holds promising applications in diagnostic techniques, imaging technologies, and bone implants [51]. It should also be noted that the subcutaneous implantation of Ga metal or alloy in guinea pigs led to necrosis at the implantation site [52].

### 3.3. Chromium

Chromium (Cr) is instrumental in reducing blood sugar levels and enhancing insulin function. It is known that, in patients with diabetes, the concentration of Cr tends to decrease. Additionally, Cr plays a vital role in augmenting insulin action, improving carbohydrate metabolism, and reducing insulin resistance [53,54,55]. Moreover, the corrosion and adhesion outcomes, coupled with the minimal release rate of Cr ions into Ringer’s solution, could pave the way for advancements in the assessment of immune cell activation and other biocompatibility tests, which are crucial for diverse medical implant applications of Cr oxide coatings [56]. The toxicity of Cr is generally linked to its valence state. Thus, Cr^6+^ exhibits strong mobility and toxicity, possessing a higher membrane penetration ability than Cr^3+^, thereby posing a greater risk to the human body. Furthermore, Cr^6+^ is known to have carcinogenic, reproductive toxic, and mutagenic effects [57].

### 3.4. Barium Titanate/Barium Strontium Titanate

Lead-free piezoelectric materials like barium titanate (BaTiO_3_, BT) and their composites, pertaining to the x[Ba(Zr_0.2_Ti_0.8_)O_3_]-(1-x)[(Ba_0.7_Ca_0.3_)TiO_3_] (BZT–BCT) system, have been shown to enhance cells’ growth and differentiation by replicating the tissues’ natural stress-generated electric potential [58]. Moreover, HA–piezoelectric material composites have been demonstrated to enhance electrical properties, accelerate cell growth, and consequently, improve the mechanism of bone repair [59,60,61,62].

It should be emphasized that BaTiO_3_ has been extensively researched due to its uncomplicated crystalline structure, mechanical and chemical stability, excellent ferroelectric properties, and high dielectric constant [63,64] (i.e., ~6000, for a fine grain size of <1 µm, and 1500–2000, for a coarse grain size of >1 µm). Notably, it possesses a Curie temperature ranging from 120 to 130 °C, which represents a factor of significant importance for its ferroelectric characteristics [65].

Composite materials of piezoelectric BaTiO_3_ with HA have demonstrated chemical resemblance to bone mineral content and exhibited responsiveness to mechanical stress. Moreover, due to the piezoelectric nature of BT, the growth of osteoblasts and lack of cytotoxicity on BT–HA-based bilayer coatings has been demonstrated [66,67]. In addition, the incorporation of Sr into the BaTiO_3_ structure enhanced the proliferation rate, differentiation, and biomineralization of osteoblasts [68]. Cytotoxicity tests have also revealed that the ion release from BT material surfaces had only a slight impact on the viability of L929 fibroblasts [69]. Data collected from animal studies suggest that elevated uptake levels of Ba^2+^ (150–450 mg/kg/day) are associated with high blood pressure, kidney and liver failure, the stimulation of smooth, striated, and cardiac muscles, and disorders of the central nervous system [70].

### 3.5. Zinc

Zinc (Zn), a trace element present in small quantities in the human body (specifically in enamel and bone [71]), is considered essential due to its proven bioactivity and strong biocompatibility. Serving as the active center for over 200 enzymes in the human body, Zn plays a crucial physiological role in bone formation and mineralization. It also influences the critical size of apatite lattice during the nucleation phase [72]. The addition of Zn^2+^ has been reported to positively impact cell functionality by enhancing the activity of alkaline phosphatase [73]. Zn-doped HA has been confirmed as an effective antimicrobial agent against both Gram-positive and Gram-negative bacteria commonly found at implant sites, including *S. aureus* and *E. coli*. Thus, the release of Zn^2+^ ions demonstrated efficacy against fungal infections, with a significant reduction in 72 h *C. albicans* biofilms observed at a Zn concentration of 3 at.% [74]. Zn-doped HA structures demonstrated an adaptable degradation rate and demonstrated both in vitro and in vivo biocompatibility, making them suitable for use as orthopedic implants [75]. It is important to mention that Zn^2+^ possesses a smaller ionic radius than Ca^2+^, enabling it to replace Ca in the HA structure, resulting in improved characteristics, such as enhanced antibacterial effects and excellent promotion of cell viability. But, in certain scenarios, the introduction of Zn into HA powders exhibited a toxic impact on cells, specifically human hepatocytes cells [76], which was attributed to the sedimentation of Zn–HA particles onto the cells.

### 3.6. Silver

Silver (Ag) ions are of significant interest, especially when used for medical purposes, due to their low toxicity to human cells and high thermal stability. They have been found to have extensive uses in medical devices such as prostheses, catheters, and skin applications. Notably, Ag ions are renowned for their ability to prevent bacterial colonization [77] and are commonly used as an essential doping element for various biomaterials [78,79,80,81,82], including their incorporation into HA [83]. Thus, the presence of Ag nanoparticles (AgNPs) has been shown to completely eliminate bacterial strains, with the bactericidal properties attributed to the release of Ag ions from the particles [84]. However, it is important to highlight that there has been limited investigation of the electrical and dielectric transport properties of Ag-doped HA, particularly in the realm of drug carrier applications [85]. Studies on in vivo toxicity and biodistribution have demonstrated the translocation, accumulation, and toxicity of AgNPs in various organs (i.e., lung, liver, kidney, brain) [86].

### 3.7. Magnesium

Magnesium (Mg) is a vital element for various physiological processes, including the development of robust bones and artificial bone substitutes [87]. Within the HA lattice, Mg is commonly used as a cationic substituent. Studies have demonstrated that an increase in Mg content resulted in a reduction of lattice size [88]. Also, higher Mg concentrations in HA determined a reduction in the crystallinity degree. Mg ions play a supportive role in the function of osteoblasts and osteoclasts, both of which are pivotal in the process of bone mineralization. The cell viability of MG63 cells has been demonstrated to be significantly enhanced by minimizing the concentration of released Mg^2+^ ions through the deposition of hydrothermal HA and Sr-doped HA coatings [89]. It is worth mentioning that a deficiency in Mg can lead to osteoporosis [90].

### 3.8. Strontium

In recent years, Strontium (Sr) ions have demonstrated their beneficial role in various bone-related applications, including the inhibition of osteoclast differentiation [91,92,93,94], stimulation of bone formation [95], and improvement of bone’s mechanical properties [96,97]. Sr represents an important element for the human body, with 98% of its total concentration being found both in the body’s skeleton and bone tissues. It is important to mention that the physical–chemical structures of Sr and Ca are similar, and the mechanism of Sr accumulation in bones can be summarized as the following: after the administration of Sr salt for preventing and treating osteoporosis, Sr diffuses through capillary walls to reach extracellular bone fluid, where it accumulates on the surface of bone apatite or replaces Ca in the bone structure [98,99]. Tetra-calcium phosphate cements have been demonstrated to exhibit superior antibacterial activity compared to the control against both *S. aureus* and *E. coli* after 7 days of immersion. This effect was attributed to the high and sustained release of Sr^2+^ ions [100]. Considering these factors, it can be concluded that Sr could serve as an excellent doping agent for HA [101]. It was also reported that prolonged exposure to elevated levels of Sr can result in decreased bone mineral density, ultimately leading to significant osteoporosis [102].

### 3.9. Vanadium

Vanadium (V) has been reported to promote bone formation through its mitogenic effect on bone cells and exhibit antitumoral activity [103,104]. Additionally, studies have demonstrated that the V ions perform as electron receptors within the HA lattice [105]. It has been reported that V ions may induce a high content of crystal defects within the HA lattice, potentially promoting an accelerated degradation process. The dissolution of V-doped HA is anticipated to release these ions into the surrounding media. As a result, the antibacterial effect of V ions is anticipated to be notably pronounced [106]. One should note that V salts (e.g., VOSO_4_·5H_2_O or Na_3_VO_4_) were reported as being unsuitable as drugs because of their poor bioavailability and notable toxicity [107]. Additionally, V complexes with organic ligands have proven unsatisfactory for oral administration due to their instability under gastrointestinal conditions [108].

### 3.10. Copper/Copper Oxide

In comparison to other metallic ions, such as Ag, copper (Cu) ions have demonstrated increased antibacterial activity [109,110]. In addition to cost-effectiveness and thermal stability, copper oxide (CuO) also has excellent optical and electrical properties [111,112]. Inside the human body, CuO has several essential roles, including (i) maintaining bone volume, (ii) accelerating wound healing, (iii) facilitating enzymatic reactions, (iv) aiding in nucleic acid formation, and (v) strengthening the immune system. Furthermore, CuO exhibits powerful antioxidant, anticancer, and antibacterial properties [113,114], making it a promising candidate for orthopedic applications [115]. It is also important to mention that the formation of soluble CuO in the aqueous phase, followed by the release of Cu ions from these oxides, constitutes key factors contributing to the antimicrobial properties of Cu [116]. It has also been reported that the elevated surface area of CuNPs renders them susceptible to oxidation [117].

### 3.11. Nickel

It has been reported that the concentration of Ni inside the human body is ~0.1 ppm [118]. Due to some important properties (i.e., anticorrosive, antibacterial, and mechanical), Ni has been found to have many applications in the biomedical field [119,120,121]. Therefore, it has been demonstrated that the addition of Ni has a positive impact on the structural properties of HA [122]. It has also been shown that the release of Ni^2+^ ions resulting from corrosion plays a significant role in causing chromosome damage to bacteria [123]. Moreover, allergies and inflammations in the skin and mucous membranes, often attributed to Ni, were also reported [124].

### 3.12. Tantalum Pentoxide

Tantalum pentoxide (Ta_2_O_5_) coatings have gained prominence in the biomedical field [125] due to their outstanding characteristics, including high corrosion resistance and biocompatibility [126], osseointegration ability [127,128], bioactivity [129,130], increased wettability [126,131,132], exceptional durability and hardness [133], and good wear resistance [127]. Moreover, it has been demonstrated that the release of Ta ions plays a pivotal role in promoting osteogenesis [134]. However, Ta has also been reported to induce the partial decomposition of HA into β- and α-tricalcium phosphate [131].

### 3.13. Alumina

Alumina, also known as aluminum oxide, is renowned for its chemical stability and superior mechanical properties. Thus, its high compressive strength and inert behavior make it a viable choice for orthopedic joint prostheses [135]. Subsequent data collected at 2 to 5 years post-implantation surgery revealed that the bone adjacent to alumina-coated stems exhibited demineralized lamellar and Haversian structures. This phenomenon was attributed to the release of Al^3+^ ions from the alumina coating [136].

### 3.14. Lithium

Lithium (Li), which is found in trace amounts in the human body, is an alkali metal known for its bioelectric properties [137]. Li, an economical drug, has primarily been employed in hematology and psychiatry, particularly for the treatment of bipolar disorders. Reports indicate that the introduction of Li+ results in the enhancement of mechanical characteristics [138], coupled with effective antimicrobial capabilities [139] in HA-based materials. Furthermore, the stimulatory effects of Li on the proliferation of human cells have also been reported [140]. Complementary studies have demonstrated that the release of Li^+^ ions contributes to an improvement in the biological response of implanted materials. This enhancement was reflected in the growth, proliferation, and differentiation of osteoblast cells through the stimulation of the Wnt signaling pathway [141] and resulted in enhanced bone regeneration in vivo [142]. Despite its established efficacy, the administration of Li was associated with both short-term side effects (such as thirst, excessive urination, nausea, weight gain, sexual dysfunction, and dermatological effects) and long-term effects on the thyroid and parathyroid glands, as well as the kidneys [143]. Nevertheless, recent studies have challenged the negative perception of Li administration, contending that intoxication occurs only when the concentration exceeds 1.5 mmol/L. Moreover, the main adverse effects can be adequately monitored and managed [139].

### 3.15. Iron

Iron (Fe) is a trace element in the human body, and Fe–HA has been found to have applications in various fields, such as drug delivery, medical imaging, and hyperthermia-based cancer therapies [144,145]. The development of magnetic HA (Fe– or FeO–HA) nano-biocomposites is of significant interest because the presence of Fe or FeO can enhance the stability of HA layers [146]. In this regard, the concentration of Fe plays a crucial role in both the magnetic properties and bioactivity of HA [147]. Clinical studies have demonstrated a significant relationship between Fe and bone density [148]. There are reports indicating that the leaching of Fe^3+^ ions from doped HA promoted the proliferation of human osteoblast-like cells [149].

## 4. Polymer/Hydroxyapatite Blends

HA has been demonstrated to undergo ion exchange reactions, allowing for the (i) incorporation of various dopants, such as metals and nonmetal ions, and (ii) formation of blends using different polymers to enhance its functionality. Polymers, owing to their versatile structure, have found diverse applications in sensors, biophysics, electronics, and medicine [150].

### 4.1. Polymethylmethacrylate

Polymethylmethacrylate (PMMA) has gained widespread usage in biomedical applications due to its excellent resistance to degradation and insulating properties for heat and electricity [151], as well as its lightweight nature, ease of polishing and processing, and cost-effectiveness [152]. It has extensive applications [153], particularly in bone cement, for filling spaces between implants and bones.

### 4.2. Polyvinyl Alcohol/Carboxymethyl Cellulose

Biopolymers, such as polyvinyl alcohol (PVA), cross-linked PVA, and carboxymethyl cellulose (CMC), are generally used for medical applications due to their hydrophilic nature, lack of toxicity, and biodegradability [154,155]. Moreover, both simple and doped PVA-based materials have been demonstrated to possess significant storage capacity, high dielectric strength, and excellent optical and electrical properties [154,156].

### 4.3. Chitosan

Some key attributes, such as chemical similarity to biological molecules, tissue compatibility, bio-resorbability, antibacterial properties, hemostatic characteristics, and the ability for chemical modifications and interaction with HA, make chitosan (CS) an appropriate solution for tissue engineering applications [157,158,159,160,161,162,163,164].

### 4.4. Conductive Polymers

Conductive polymers (CPs) are acknowledged as a category of organic materials possessing distinctive electrical and optical properties akin to those found in inorganic semiconductors and metals. The past decade has witnessed substantial research interest in CPs, including polyacetylene, polythiophene, polypyrrole, polyphenylene, polyaniline, etc. [165,166,167]. A variety of methodologies and innovative advancements have emerged for modifying and tuning CPs, facilitating their integration into diverse fields of biomedicine, including bioengineering and regenerative medicine. It should be emphasized that these materials hold the potential to establish the groundwork for future breakthroughs.

## 5. Electrical and Dielectric Properties

The physical–chemical, mechanical, and biological properties of HA-based materials have been extensively investigated for a wide range of biomedical applications. However, limited attention has been dedicated to the in-depth characterization of the electrical and dielectric characteristics of these structures. In this respect, to conduct a relevant comparative study, the introduction and comprehensive discussion of HA results will be organized based on their reported form: (i) powders/nanoparticles, (ii) pellets, and (iii) thin films. Before introducing these results, the reader will be familiarized with some general information related to the electrical and dielectric parameters that will be discussed in this review, including impedance, conductivity, dielectric constant and loss, and permittivity.

### 5.1. Impedance

In the case of HA-based materials, electrochemical impedance spectroscopy is generally used to investigate the transport phenomena of charge carriers within HA compounds [168]. Complex impedance spectra are usually applied to analyze the real electrical and dielectric nature of a material, by representation of appropriate complex equivalent circuits [169]. Impedance analysis precisely indicates the process of dielectric relaxation inside an ionic material after the application of a varying electric field [85]. Thus, studies on impedance spectra consider the interaction of an external electric field with the permeability and electric dipole moment of a specific probe [170].

### 5.2. Conductivity

It is well-known that the complex conductivity of materials consists of two components, i.e., alternating current, ac (*σ_ac_*), and direct current, dc (*σ_dc_*), conductivities. Ac conductivity is generally used to study the frequency-dependent conductive behavior of ions present in both undoped and doped HA lattices. It should also be mentioned that the *σ_ac_* arises from the mobility of ions near the surface of ionically conducting materials when they are subjected to high-frequency AC currents. This phenomenon leads to an increase in the effective resistance of a conductor [171].

A conductivity spectrum is commonly employed to analyze the dynamic behavior of charge carriers within a certain material. Changes in the impedance data can also be elucidated through a logarithmic plot of conductivity in function of the applied frequency [169].

The universal dielectric response law for complex conductivity is provided by [172]:

*σ′(f) = σ_dc_ + σ_ac_(f) = σ_dc_ + αf^s^* (1)

where *σ′(f)* represents the total conductivity, *σ_dc_* represents the dc conductivity (a plateau region of the total conductivity curve, predominant at lower frequencies due to the effective high resistance of grain boundaries), α is the temperature-dependent constant, and *s* < 1 is the frequency exponent. Conversely, the ac component becomes prominent at higher applied frequencies, indicating the dominance of long-range ionic conduction through grains. At lower applied frequencies, carriers move from one unoccupied site to another through thermal excitement, which gives rise to *σ_dc_*. At higher applied frequencies, carriers undergo thermal relaxation due to the back-and-forth oscillating ac field [85].

To calculate the *σ_ac_* of fabricated structures, the Jonscher’s equation is generally used [173]:

*σ_ac_ = σ_dc_ + Bω^s^* (2)

where *B* is a constant, *ω* is the angular frequency, and *s* is an exponent [170]. It is important to note here that the *s* values can be extracted from the slope of the *ln σ_ac_* vs. *lnω* graph lines.

The application of AC electric fields could induce modifications in the polarization of a material under investigation. Considering HA’s ionic properties, the dipole moment of the (OH^−^) group also contributes to dielectric relaxation processes [174]. Thus, *σ_ac_*, which is dependent on the applied frequency, is therefore associated with the dielectric relaxation of non-free charge carriers influenced by the AC electric field [101].

At low applied frequencies, it has been indicated that materials exhibit constant conduction values due to large polaron conduction. In contrast, the low values of polaron conduction indicate an increased conductivity. The conductivity increase with the applied frequency could be due to either proton jumping of O^2−^ or the interaction of OH^+^ ions bonded with the PO_4_ group [10]. In the case of the structures with a doping rate exceeding 2%, the conductivity was indicated to exhibit increased values [175].

### 5.3. Dielectric Analysis

From a biomedical point of view, dielectric properties are important for understanding a material’s ability to store charges under an alternating electric field. Enhancing wound healing capabilities involves the introduction of a biocompatible ceramic layer to bone implants. The significance of the electrical and dielectric properties of HA is highlighted by the demonstrated stimulation of bone fracture healing in human bodies through electromagnetic fields [170]. Ceramic materials, including HA, polarize under applied electric fields, serving as a source of electric field even after the external one is eliminated. This internal generation of an electric field within the ceramic layer continues to boost wound healing [176]. Therefore, the larger the value of dielectric constant (*ε*_1_), the stronger the generated field. As a consequence, implants covered with a biocompatible layer presenting a high *ε*_1_ value are generally preferred for clinical applications [33].

It is important to mention that the *ε*_1_ is highly sensitive to water content and the frequency of the applied electric field, as observed in measurements conducted on both human and bovine cortical bones. Interestingly, the *ε*_1_ value of dry bone is comparable to that of sintered HA [177]. The differences of the biological properties are mainly determined by the surface properties, and the *ε*_1_ parameter is related to characteristics like density and grain size [178].

When subjected to an alternating voltage of specific frequency, a dielectric material generally absorbs energy. However, due to its capacity limitations, a portion of its energy is stored, and the rest is inevitably released as heat at specific frequencies. The energy lost in this process is known as dielectric loss, and it is represented by *tan*(*δ*), where “*δ*” represents the phase angle.

At low applied frequencies, polarization is primarily influenced by charge carriers, requiring more energy to rotate dipoles, thus leading to higher dielectric loss. [179]. It has been reported that dielectric loss is an inherent property of dielectric materials, with minimal dielectric loss indicating a higher-quality material [171].

It should also be mentioned that *tan*(*δ*), which indicates energy loss associated with the dielectric relaxation process, is defined as the ratio between the imaginary part (*ε″*) of the permittivity (*ε*) and the real part (*ε′*) [101]:

*tan*(*δ*) = ε″/ε′

It has been reported that *tan*(*δ*) is inversely proportional to applied frequency [180]. This behavior is attributed to the hopping process of free-charge carriers in the low frequency domain, leading to an increase in the *tan*(*δ*) values [181].

### 5.4. Permittivity

Permittivity (*ε_r_*) represents the ability of dielectric materials to store energy and signifies the magnitude of dipole alignments, indicating total polarization [169]. The dielectric permittivity of a material offers essential information on the extent of polarization within specific grains and grain boundaries, regulating the surface potential difference in nanostructures. This information is crucial for drug delivery applications [85].

### 5.5. Simple and Doped Hydroxyapatite Powders/Nanoparticles

Table 1 introduces information related to the used materials, synthesis techniques, and tackled electrical parameters in the case of HA-based powders/nanoparticles. For more detail on the studies, refer to the corresponding references provided in the last column of Table 1.

#### 5.5.1. Impedance Spectroscopy

The impedance values inferred for the samples investigated by AL-Wafi et al. [97] have been used to explore the relationship between alternating current conductivity (*σ_ac_*) and angular frequency (ω) [185]. It has thus been demonstrated that *σ_ac_* values increase with applied ω [97].

In a complementary study conducted by Poovendran et al. [183], the impedance values of the investigated samples were shown to drastically decrease with an increase in the applied frequencies. Moreover, impedance studies revealed that with an increase in temperature, the values of capacitance and dielectric constants progressively decreased [183].

Furthermore, it has been reported that impedance values decrease with increases in applied frequencies and temperature [26]. This observation indicates that the material’s dielectric relaxation is dependent on both temperature and the imaginary part of the impedance (*Z*″), which merged at high frequencies due to the accumulation of space charges. Moreover, the real part of impedance decreased with an increase in temperature, which indicates high conduction in the materials due to the presence of grains. The arbitrary modification in impedance with the applied temperature were attributed to inhomogeneity. Relaxation peaks have been observed in the low-frequency region, while in the case of higher frequencies, these peaks were shown to shift. This phenomenon is a clear indication of the presence of thermally activated charge carriers at both high frequencies and temperatures [26].

#### 5.5.2. AC Conductivity

When plotting alternate current conductivity (*σ_ac_*), Yahia et al. [182] found that its value increased with the applied frequencies, for both simple and doped samples, following the universal power law. The inferred values corresponding to the frequency exponent *s* fell within the range of 1 to 1.008 for both type of samples (i.e., simple and doped). These values were similar to those reported in the literature for ionic conducting materials [186], and the theoretical limit of one. The *s* values were shown to increase at low Te concentrations, whereas at higher concentrations, they resembled those corresponding to undoped HA. Moreover, the inferred values of the dielectric constant and *σ_ac_* were observed to be influenced by the doping agents. These results aligned well with previous findings [20,187].

It has been shown that the lowest *s* value is obtained in the case of the highest Sr doping concentration (i.e., 0.48), while the highest *s* value is obtained at a value of Sr doping of 0.24. This modification of the *s* values with the Sr doping concentration has been attributed to the existence of a new phase (i.e., nanostructured strontium apatite) [97].

The *σ_ac_* values have been indicated to increase with the applied frequencies [7]. Badran et al. [7] demonstrated the modification of the *s* values with Li concentration in the investigated structures. The observed behavior was similar to that reported in the work of Kaygili and Tatar (2012) [188], where it was indicated that the differences in the *s* values were due to the interaction between HA and Li-dopant ions influencing the process of ac transport [7]. The *σ_ac_* values were similar for the synthesized particles, which varied with Ag doping rate and increased with the applied frequencies [7].

In another study, it was demonstrated that the maximum *σ_ac_* values were inferred at increased V concentrations (i.e., 30% V) [105]. By examining the slopes of the linear portions, the frequency-dependent exponent (*s*) can be determined for samples with varying V concentrations. The *s* values are depicted as 0.9942, 0.9985, 0.9947, 1.0023, 1.0105, and 1.0089, corresponding to pristine, 1, 5, 10, 20, and 30% V, respectively [105]. One should note that the *s* values typically range from 0 to 1, with an ideal Debye-type sample having a value of 1. Thus, the presence of defects and charge carriers in the substance leads to variations in *s* values, which is attributed to the influence of charge carriers and changes in extrinsic dipole.

In the case of Fe-based HA structures doped with Cu in various concentrations, it was observed that the *σ_ac_* values remained constant with increasing frequency up to 10^5^ Hz; beyond this threshold, *σ_ac_* values increased with the applied frequencies, following the Jonscher universal power law [189]. The inferred *s* values were less than unity, which corresponded to the impossibility of a direct measurable current conductivity. Thus, the motion of the fabricated structures indicated abrupt hopping with translational motion [190,191,192]. When applying various DC bias voltages, in all investigated samples, no changes in the *σ_ac_* values up to 10 MHz were observed [170].

When using high frequencies, Younes et al. [115] showed that *σ_ac_* values had a continuous increase due to ions’ segregation along the *c*-axis of the HA crystal structure [2]. When increasing the HT and CuO contents, the *σ_ac_* values increased continuously due to the increase in the number of charge carriers. This consequently reduced nanocomposite resistance [156,193]. In the same study, it was reported that the formed apatite layer slightly increased the *σ_ac_* values [115]. This could be related to the fact that the formed HA layer reduced the number of surface pores, which subsequently diminished the *σ_ac_* values. In comparison, other fabricated samples were negatively affected by this HA layer developing onto the surface. This was explained both by the fact that the HA layer hinders the mobility of the conducting CuO and HT ions between the two electrodes and that the HA value of the electrical conductivity is lower than the one corresponding to CuO and HT [115]. Another important result of this study was related to a significant improvement in the electrical conductivity of the samples with an increase in both frequencies, as well as CuO and HT contents [115].

When dealing with polymeric structures, the *σ_ac_* values have been shown to increase with the applied frequencies. It should be noted that the *σ_ac_* value of a PVA/CMC blend inferred at RT and 1 kHz was 8.81 × 10^−8^ Ω^−1^m^−1^. After adding 40 wt.% AgHA–NPs, a significant increase in this value up to 1.84 × 10^−7^ Ω^−1^m^−1^ was reported [154]. It has also been shown that the conductivity logarithm *σ*(*w*) of the investigated polymeric blend increases almost linearly with the applied temperature, AgHA–NPs doping, and frequencies [154].

**Figure 1:**
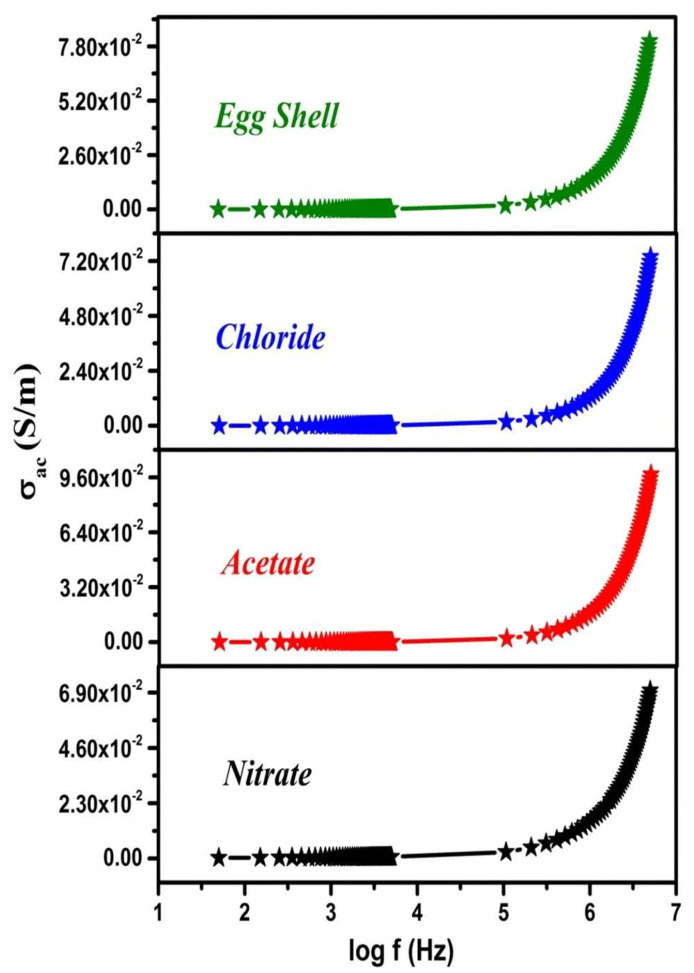
Alternating current conductivity (*σ_ac_*) vs. frequency (*f*) plots of the as-synthesized hydroxyapatite samples [184].

In their study, Sundarabharathi et al. [184] investigated the behavior of the *σ_ac_* of HA NPs fabricated using different anionic precursors as a function of the applied frequencies (Figure 1).

In Figure 1, it can be seen that the values of *σ_ac_* gradually increased with the applied frequency at RT for all the investigated HA samples and obeyed the universal power law [194].

#### 5.5.3. Dielectric Analysis

##### Dielectric Constant

**Figure 2:**
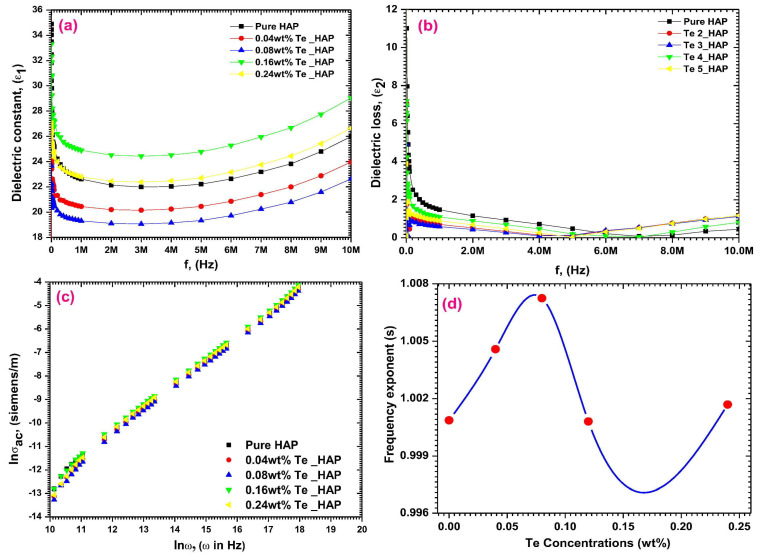
Plots of variation of (**a**) dielectric constant (*ε*_1_), (**b**) dielectric loss (*ε*_2_), (**c**) ac conductivity (*σ_ac_*), and (**d**) frequency exponent (*s*) for undoped and Te-doped hydroxyapatite nanostructures. Reproduced with permission from [182].

Figure 2 illustrates the plot depicting the variation in the dielectric constant (*ε*_1_) values with the applied frequency at room temperature (RT) (Figure 2a), the dielectric loss (*ε*_2_) (Figure 2b) and *σ_ac_* (Figure 2c) depending on the applied frequency, and the inferred values of *s* (Figure 2d). It is evident that *ε*_1_ is frequency-dependent, and all samples exhibited similar behavior.

At low applied frequencies, the *ε*_1_ values were shown to decrease with increasing frequency until it reached 1 MHz and remained relatively stable between 1 and 5 MHz (Figure 2). The existence of high *ε*_1_ values at these low applied frequencies could be related to the influence of electronic, ionic, dipolar and space charge polarizations [195]. When doping HA with Te concentrations of 0.16 and 0.24 wt.%, the *ε*_1_ values enhanced, whereas lower concentrations (i.e., 0.04 and 0.08 wt.%) resulted in decreased values. This modification of the *ε*_1_ values was linked to Te ions inducing significant dielectric polarization. This consequently facilitated the spread of electromagnetic fields to aid in healing bone fractures [182]. One should note that these results align well with previous studies on HA doping with Cd [12], the obtained *ε*_1_ values being even higher than the ones observed in the case of Fe-doped HA [14,20].

**Figure 3:**
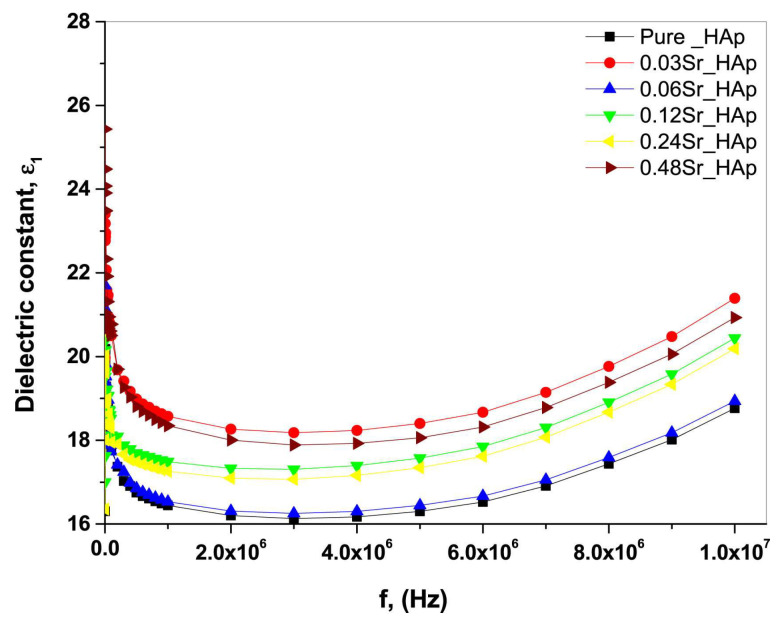
The relation between the dielectric constant (*ε*_1_) and the frequency (*f*) of undoped and Sr-doped hydroxyapatite nanorods. Reproduced with permission from [97].

The *ε*_1_ values in the case of simple and Sr-doped HA samples fabricated by AL-Wafi et al. [97] in various concentrations were inferred from capacitance measurements of the investigated structures. Figure 3 displays the correlation between the external frequency of the electrical field and *ε*_1_ for all fabricated samples.

At frequencies below 5 MHz, it was found that *ε*_1_ values remained relatively stable, while at higher frequencies, they exhibited an increase proportional to the applied frequencies (Figure 3). It is important to note that the *ε*_1_ values were demonstrated to increase with the doping concentration of Sr in HA, which is in agreement with previous reports [196]. A possible explanation of this result is related to the generation of ionic polarization induced by electric dipoles [187,197]. Moreover, the *ε*_1_ values for the various Sr doping concentrations (i.e., 0.03, 0.06, 0.12, 0.24, and 0.48), were consistently higher than those corresponding to pristine (undoped) HA. This result was correlated with an improved phase crystallinity in comparison with simple HA [97]. Usually, the modification of the *s* parameter values with the variation of the doping concentration in the HA matrix could be defined by changes in the *ε*_1_ values influenced by the polarizability of the applied AC field in the matrix.

**Figure 4:**
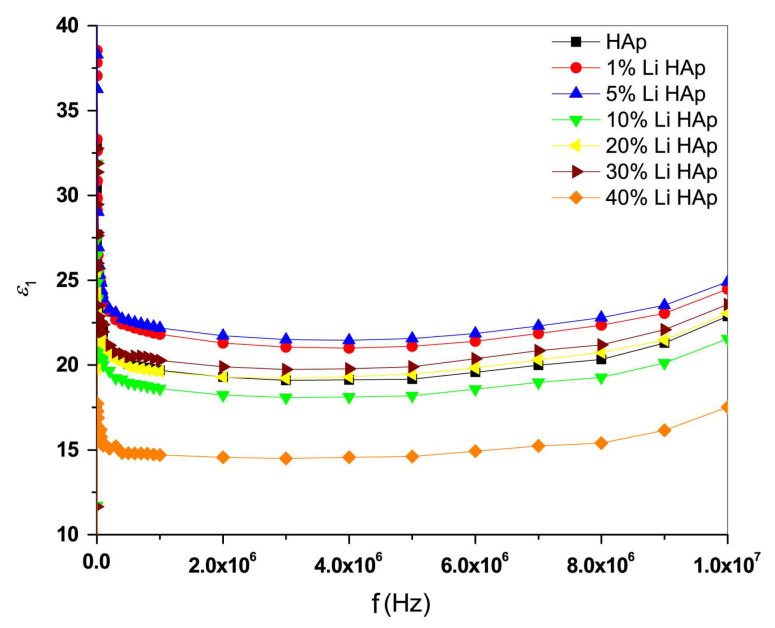
The frequency (*f*) dependence of the dielectric constant (*ε*_1_) of undoped and lithium-doped hydroxyapatite nanocomposites. Reproduced with permission from [7].

In their study, Badran et al. [7] investigated the correlation between applied frequencies and *ε*_1_ values in the case of undoped and Li-doped HA nanocomposites at RT (Figure 4).

At frequencies below 0.5 × 10^6^ Hz, *ε*_1_ values were shown to decrease as the applied frequency increased, while at frequencies above 0.5 × 10^6^ Hz, the changes in *ε*_1_ values were insignificant (Figure 4). This behavior was in accordance with the finding that at low applied frequencies, the polarization phenomenon closely follows variations in the applied field. However, at higher applied frequencies, the dipole moment of OH^−^ ions could not keep pace with the field changes, consequently leading to a decrease in polarization and *ε*_1_ values [7,198]. Moreover, the Li concentration was reported to influence *ε*_1_ values. Thus, Kaygilia et al. [137] have demonstrated that Li-doped HA structures with low *ε*_1_ values present advantages for bone fracture healing. Considering these findings, one can conclude that the reported results on various concentrations of Li-doping in HA advance the application of this biomaterial in dental and orthopedic implants [7].

**Figure 5:**
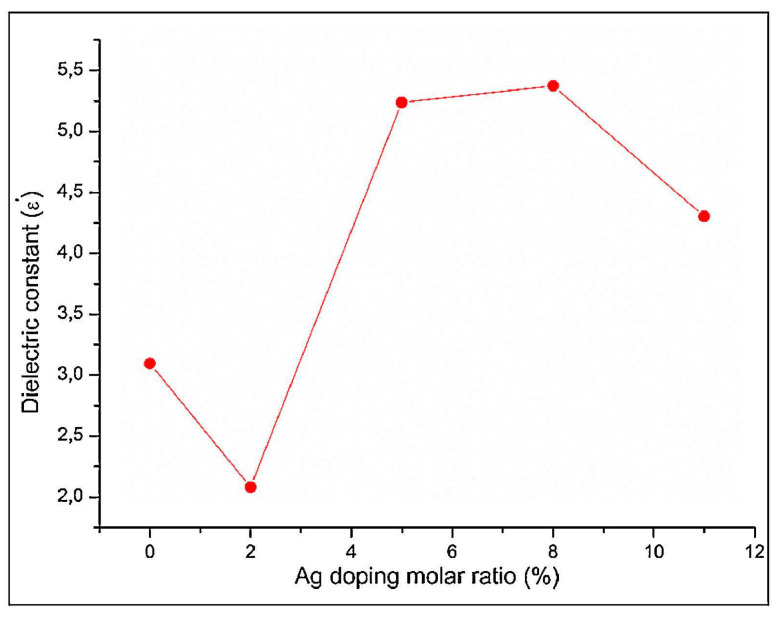
The variation of the dielectric constant (*ε*’—referred to as *ε*_1_ in the text) vs. silver doping ratio at 1 kHz. Reproduced with permission from [175].

**Figure 6:**
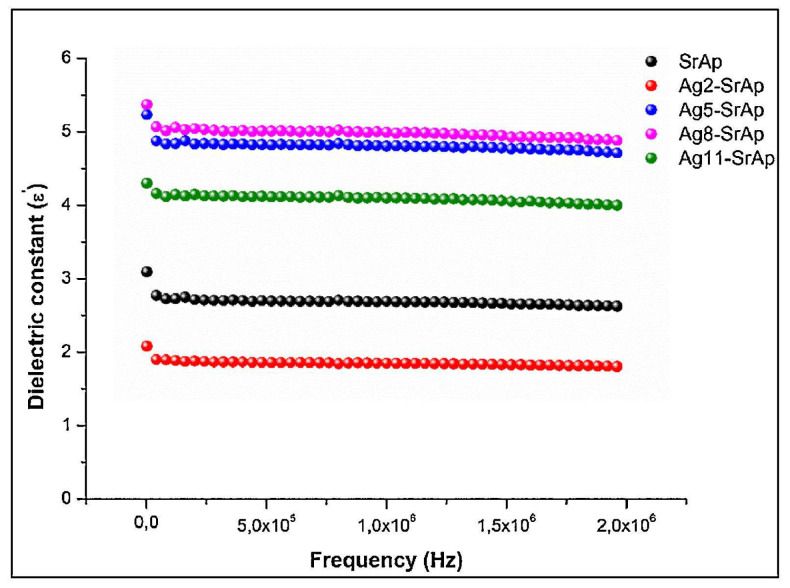
The variation of the dielectric constant (*ε*’—referred to as *ε*_1_ in the text) vs. frequency of synthesized particles. Reproduced with permission from [175].

In another study [175], it was demonstrated that the *ε*_1_ values of the fabricated strontium apatites (SrAp) were influenced by Ag doping (Figure 5) and slightly decreased with increasing applied frequency (Figure 6).

It has been reported that at high applied frequencies, the dipole moments of OH^−^ ions do not react to changes in the applied field. This leads to diminished polarization and, consequently, to a decrease in the *ε*_1_ values [28,198,199]. These modifications in *ε*_1_ values are related to variation in the electrical polarization of SrAp induced by Ag doping. It was observed that along with the addition of Ag ions to SrAp structure, the electrical dipole moments of OH^−^ ions, which have an ionic structure, oscillated in response to the frequency of the applied electric field. This oscillation consequently determined a modification of the *ε*_1_ values within the dipoles’ oscillations [28]. This finding is in agreement with previous studies demonstrating that doping the HA structure with various ions clearly altered the *ε*_1_ values [12,28,164,191,200]. In the case of SrAp samples, the *ε*_1_ had a value of ~3 at 1 kHz, which changed to 2–5 after Ag ion doping. This result clearly indicated that Ag doping had a substantial impact on both the *ε*_1_ values and polarizability of SrAp. The results of this study also demonstrated increased *ε*_1_ values in Ag-doped samples as compared with simple (undoped) SrAp. Considering the benefits of HA-based materials with low *ε*_1_ values in bone fracture healing [7], these Ag-doped structures could be advanced as promising candidates for dental and orthopedic implant-type applications [175].

**Figure 7:**
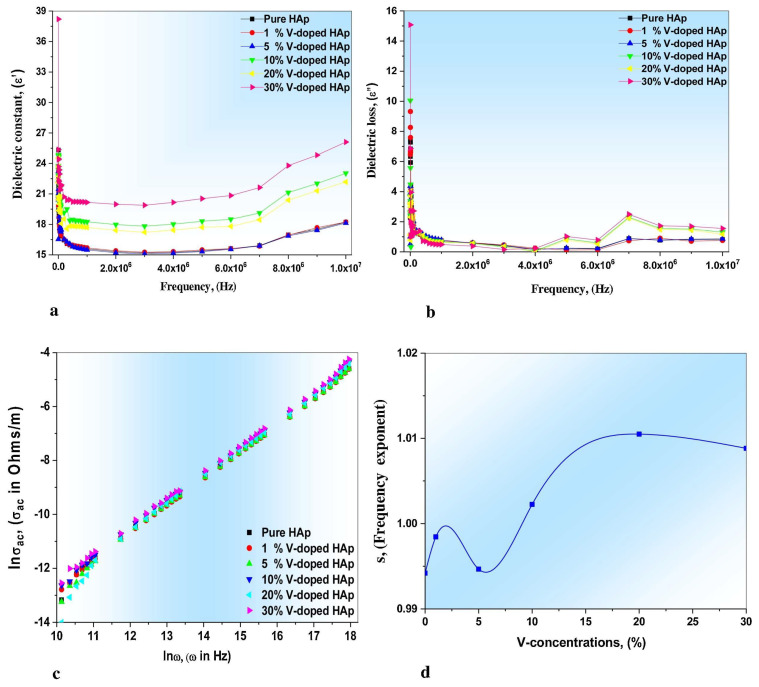
(**a**) Dielectric constant (*ε*’—referred to as *ε*_1_ in the text) and (**b**) dielectric loss (*ε*″—referred to as *ε*_2_ in the text) as a function of frequency plots for undoped and V-doped hydroxyapatite nanostructures, (**c**) alternating current conductivity (*σ_ac_*) vs. frequency plots for undoped and V-doped hydroxyapatite nanostructures, and (**d**) frequency exponent (*s*) as a function of V concentrations for undoped and V-doped hydroxyapatite nanostructures. Reproduced with permission from [105].

Rajhi and his collaborators reported on the variations of *ε*_1_ (Figure 7a), *ε*_2_ (Figure 7b), and *σ_ac_* (Figure 7c) function of the applied frequencies, along with the inferred *s* values (Figure 7d). Figure 7a shows decreased *ε*_1_ values in all examined specimens when applying high frequencies [105].

It is known that the *ε*_1_ values for simple (undoped) HA typically range from 15 to 23. Additionally, it has also been demonstrated that the *ε*_1_ values for V-doped HA structures increase proportionally with V concentration within the HA matrix. The maximum inferred values were thus observed for a V concentration of 30% [105].

Helen S. and Kumar A.R. [26] investigated the frequency dependence of *ε*_1_ in samples of different concentrations. The increased *ε*_1_ values inferred at low applied frequencies were related to the existence of diverse polarizations (i.e., space charge, ionic, dipolar, and electronic) within the doped HA matrix [201]. Moreover, the decline in *ε*_1_ values obtained when increasing the applied frequency evidenced the existence of dielectric relaxation within the fabricated samples [26].

The *ε*_1_ values have also been shown to increase with the doping concentration, while the permittivity dispersion at low applied frequencies promptly decreased with the rise in applied frequency. At the same low frequencies of the applied electric field, the values of the imaginary dielectric constant also increased [85]. It should be noted that despite the reduction in the number of ions in control for dipolar and ionic polarization in a doped system, the released OH^−^ ions remained trapped within the interior. Instead of escaping, these ions accumulated along with other charge carriers from the HA lattice within the spaces between grains and grain boundaries. As a consequence, significant space-charge polarization was produced due to the high charge accumulations in these regions, leading to a significant enhancement in the *ε*_1_ values of the doped structures [85].

**Figure 8:**
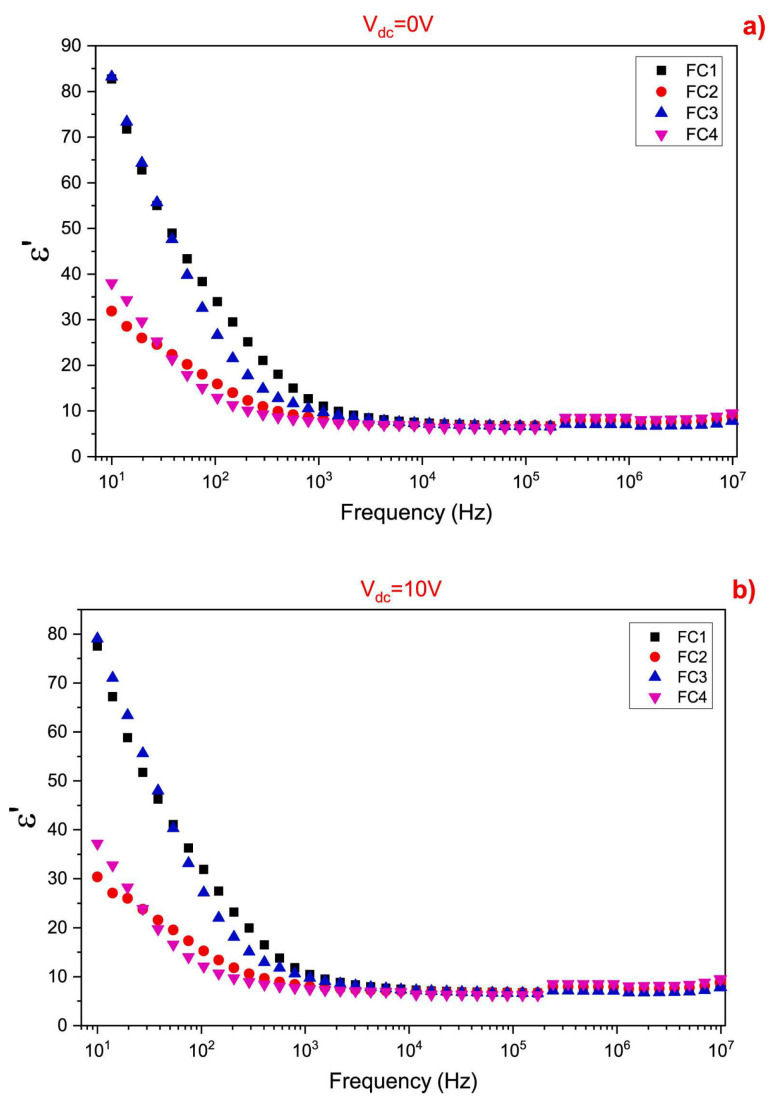
Behaviors of dielectric constant (*ε*′—referred to as *ε*_1_ in the text) as a function of frequency for (**a**) V_dc_ = 0 V and (**b**) V_dc_ = 10 V. Reproduced with permission from [170].

In their work, Ercan et al. [170] examined the behavior of the fabricated Fe-based HA structures doped with Cu in various concentrations (i.e, 0, 0.29, 0.58, and 0.87 at.%, referred to as FC1–FC4) by performing an analysis of *ε*_1_ as a function of the applied frequencies within the range of 10 Hz to 10 MHz. At zero DC bias voltage (V_dc_) and low frequencies, the calculated *ε*_1_ values were notably high (Figure 8a) [170].

When the applied frequency increased up to 1 kHz, the *ε*_1_ values rapidly decreased and persisted relatively constantly until reaching 10 MHz. The nature of the dopant in the HA matrix influenced the observed *ε*_1_ values with increasing the applied frequency. For instance, in the case of Zn-, Mg-, and Co-doping of HA samples, the observed *ε*_1_ values decreased, fluctuated, and increased, respectively [190,202]. In comparison, the findings of Ercan et al. [170] demonstrated a decrease in *ε*_1_ values with increasing frequency in Fe-based HA samples doped with Cu in various concentrations. This finding was consistent with the results reported by Panneerselvam et al. [203]. When applying a 10 V DC bias voltage, a slight decrease in *ε*_1_ values was observed (Figure 8b). It is important to mention that, when increasing the applied frequency, no significant changes in overall behavior were noticed. However, when DC bias voltages in the range of −10 V to +10 V were applied, the behavior of *ε*_1_ values exhibited a distribution at low applied frequencies, followed by a decrease with increasing frequencies. It was, therefore, concluded that further increasing the DC bias voltage determined a decline in polarization [170].

Youness et al. [115] reported that the *ε*_1_ values decreased when increasing the applied frequency. In addition, both CuO and HT contents increased in the fabricated samples. It is important to note that, up to 5 MHz, the reduction of *ε*_1_ values when increasing the applied frequency had a gradual behavior, whereas at higher frequencies, a significant decrease in *ε*_1_ values was observed. This decline could be connected to the alignment of dipoles within the fabricated structures that have a tendency to position themselves in the direction of the applied electric field. Conversely, at low applied frequencies, the inferred *ε*_1_ values were relatively high, most probably due to the slow relaxation of highly oriented dipoles [2]. Additionally, the increase in CuO and HT contents also led to a rise in dipoles’ content, further influencing *ε*_1_ values [115]. It should also be emphasized that the *ε*_1_ values indicated contrasting behavior and increased after immersion in simulated body fluid solutions [115].

**Figure 9:**
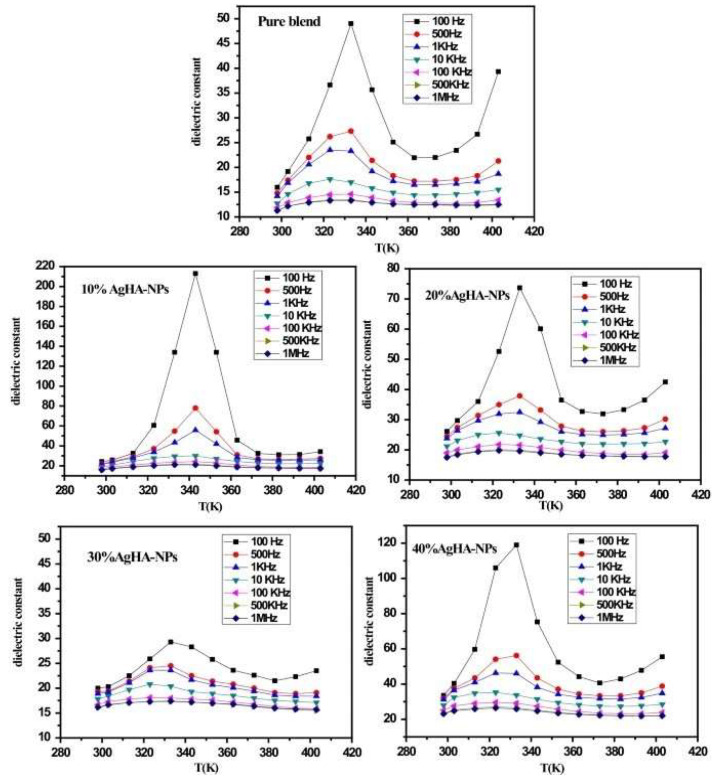
Dielectric constant as a function of temperature (*T*) of polyvinyl alcohol/carboxymethyl cellulose blend and a blend loaded with (10, 20, 30, 40 wt.%) silver-doped hydroxyapatite nanoparticles [154].

Nasrallah D.A. and Ibrahim M.A. [154] reported on the dependence of the *ε*_1_ values inferred for a polyvinyl alcohol/carboxymethyl cellulose (PVA/CMC) blend and a blend loaded with (10, 20, 30, and 40 wt.%) AgHA–NPs of different temperatures (Figure 9).

Their investigations demonstrated a consistent decrease in *ε*_1_ values when increasing the applied frequency across the frequency range of 100 Hz to 1 MHz. The plots demonstrated that at low applied frequencies and elevated temperatures, the inferred *ε*_1_ values were very high. This observation was related to possible charge accumulation within the prepared samples and the presence of permanent dipoles with sufficient time to rotate in the direction of the applied electric field [204]. Interestingly, it was also observed that beyond a critical frequency, *ε*_1_ values started to decrease until attaining constant levels for high applied frequencies. This phenomenon occurred due to the absence of ion diffusion, preventing dipole molecules from orienting toward the direction of the applied electric field at higher frequencies. Interfacial polarization was thereby eliminated [205].

In the same work, it was demonstrated that the *ε*_1_ values exhibited a progressive increase with temperature across the entire investigated frequency range [154]. This behavior could be explained as follows: at low temperatures, the thermal energy absorbed by the polymeric sample is minimal, which causes the dipoles to remain frozen and unresponsive to the applied electric field. This further results in low *ε*_1_ values. However, with the temperature rise, the viscosity of the polymeric samples decreases, providing the dipoles with sufficient energy to orient themselves effectively in the direction of the applied electric field. According to the free volume theory, this leads to a gradual increase in the *ε*_1_ values with the applied temperature, reaching a peak at 333 K [206]. It was also observed that the *ε*_1_ values increased with the temperature up to 333 K and decreased when the temperature exceeded 343 K. One possible explanation of this behavior could be related to the melting of the PVA/CMC crystalline phase at higher temperatures. Thus, a transition from a semicrystalline phase to a flow region occurred and caused a decrease in the dielectric permittivity as the contact between the AgHA–NPs and the polymer matrix reduced [207]. At lower temperatures (~320 K), the *ε*_1_ values remained relatively constant. This suggests that, at a fixed applied frequency, the thermal energy absorbed by the PVA/CMC blend was insufficient to significantly affect the motion of the polymer chains. It should be noted that an increase in temperature enhances the chain mobility of the polymer system, leading to a linear increase in *ε*_1_ up to a maximum (peak) value, beyond which they decrease with temperature. In addition to interfacial polarization at the AgHA–NPs and PVA/CMC interfaces, the inferred *ε*_1_ values were significant in the low-frequency domain. This phenomenon could be connected to both free charge motion within the polymer and the movement of polar groups [208]. Although, at high applied frequencies, the *ε*_1_ values remained small and constant, indicating that the polarized orientation and chain motion could not compete with the rapidly oscillating electric field [154]. It is also important to note that, throughout the studied frequency domain, the *ε*_1_ values of the fabricated nanocomposites were superior to those corresponding to the simple blend. Thus, at RT, the *ε*_1_ values were shown to increase with the concentration of AgHA–NPs up to 40 wt.% (reaching *ε*_1_ = 40.3 at 100 Hz) and to decrease at high applied frequencies (reaching *ε*_1_ = 24.8 at 1 MHz). Nonetheless, these values consistently remained higher than those corresponding to the simple blend (i.e., *ε*_1_ = 19.13 at 100 Hz).

**Figure 10:**
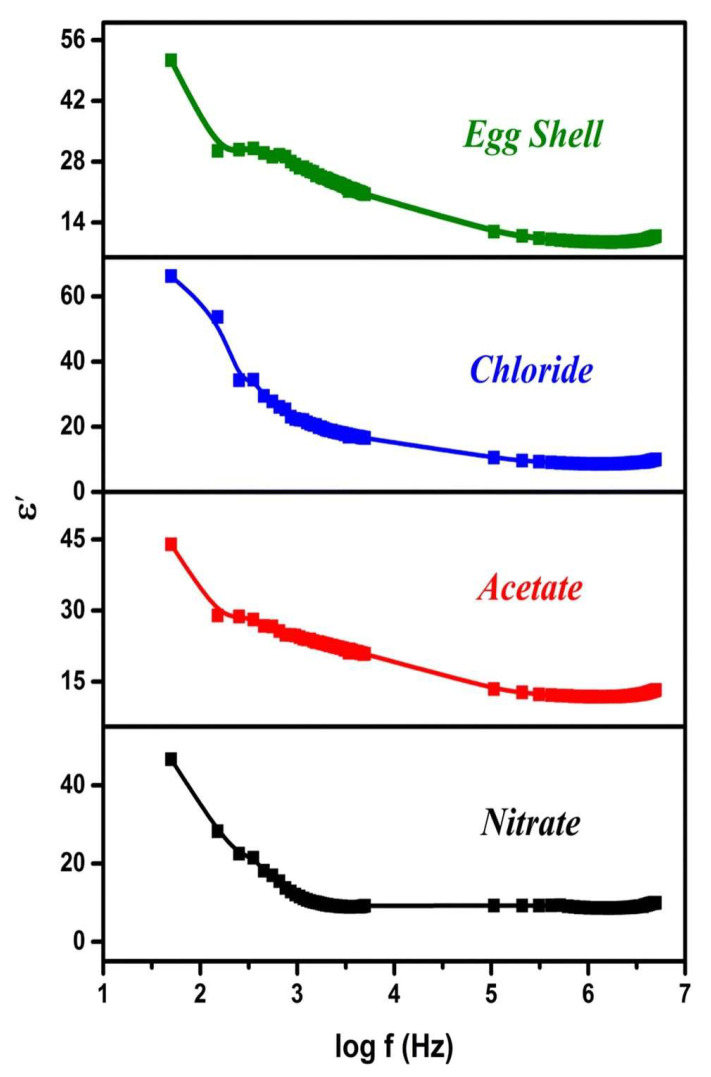
Dielectric constant (*ε*′—referred to as *ε*_1_ in the text) as a function of frequency (*f*) plots for the hydroxyapatite samples [184].

Sundarabharathi et al. [184] reported on decreased *ε*_1_ values when increasing the applied frequency of the electric field (Figure 10) in their research. This result was related to modification in dielectric polarization.

It is known that the HA nanostructure consists of an array of calcium ions in combination with phosphate and hydroxyl ions. At low applied frequencies, these ions oscillate when the natural frequency of OH^−^ bound charge aligns with the applied electric fields’ frequency. This leads to modifications in dipole moments and, consequently, affects the *ε*_1_ values at low applied frequencies. Thus, a decrease in crystallite size was associated with an increase in the *ε*_1_ values. It was observed that different anions could influence the *ε*_1_ values, which could further impact the size of the crystallites [20,209]. Additionally, changes in crystallinity were found to modify HA’s ionic polarization, resulting in a decrease in the *ε*_1_ values [210].

##### Dielectric Loss

When analyzing the dielectric loss (*ε*_2_) function of the applied frequencies (Figure 2b), a behavior similar to that observed in the case of *ε*_1_ values was reported by Yahia et al. [182]. Thus, at low applied frequencies, the *ε*_2_ values decreased with increasing frequency up to 1 MHz. However, from 1 to 5 MHz, they remained almost constant. The high *ε*_2_ values observed in the low-frequency range may be attributed to the contribution of electronic, ionic, dipolar, and space charge polarizations. The *ε*_2_ values were found to be enhanced due to Te doping in HA at higher concentrations (i.e., 0.16 and 0.24 wt.%). However, at lower concentrations (i.e., 0.04 and 0.08 wt.%), these values decreased. To conclude, the change in the *ε*_2_ values may be attributed to the presence of Te ions, which induce high dielectric polarization [182].

**Figure 11:**
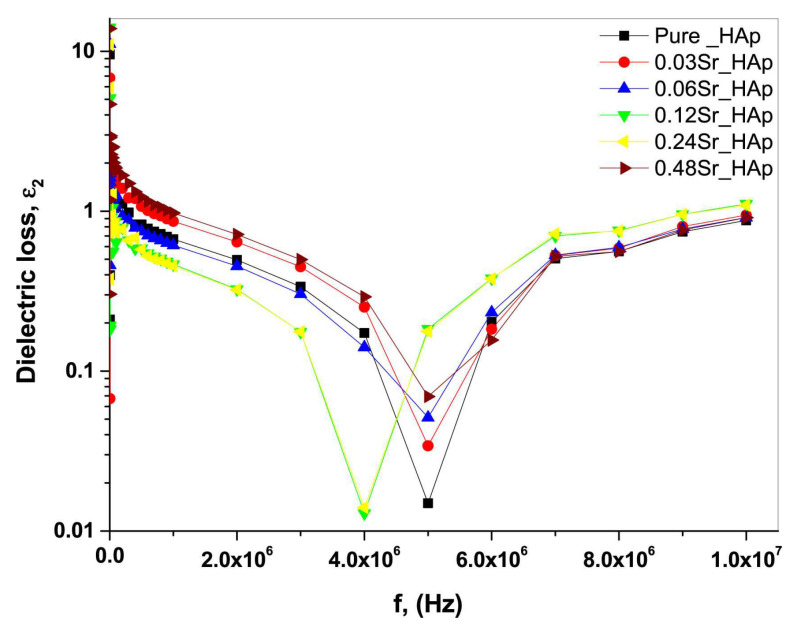
The relation between the dielectric loss (*ε*_2_) and the frequency (*f*) of undoped and Sr-doped hydroxyapatite nanorods. Reproduced with permission from [97].

AL-Wafi et al. [97] also studied the relationship between *ε*_2_ variation and applied frequency, in the case of both undoped and Sr-doped HA nanostructures, at various concentrations (Figure 11).

It was observed that the smallest *ε*_2_ value was observed at 5 MHz; beyond this frequency, the *ε*_2_ values increased, the highest value being observed at a Sr concentration of 0.12 in the HA structure (Figure 11) [97].

When investigating the frequency dependence of the *ε*_2_ at RT, Badran et al. [7] observed that it progressively decreased when increasing the applied frequency up to 1 × 10^6^ Hz. Moreover, the *ε*_2_ values showed a gradual decline as the applied frequency increased [7].

Rajhi et al. [105] revealed the presence of dielectric dispersion in the *ε*_2_ values for all their investigated samples (Figure 7b).

**Figure 12:**
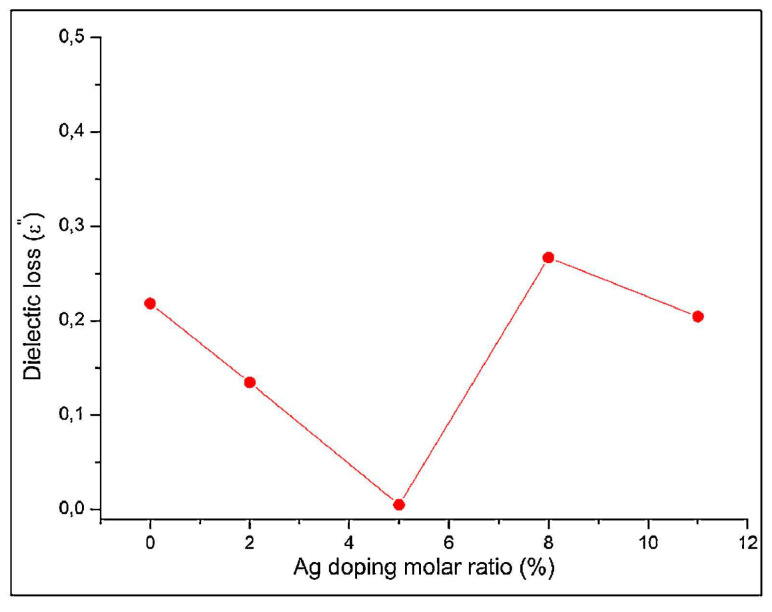
The variation of the dielectric loss (*ε*″—referred to as *ε*_2_ in the text) vs. silver doping ratio at 1 kHz. Reproduced with permission from [175].

In the case of synthesized apatite particles, the variation of the *ε_2_* values (which were calculated using Equation (2) [211]) with the applied frequency exhibited changes in response to the concentration of Ag doping. Thus, the *ε*_2_ values decreased at higher frequencies up to ~25 × 10^6^ Hz [175]. Considering the direct relationship between *ε*_2_ and energy usage, the polarization diminished because of charge accumulation, consequently leading to a reduction in the *ε*_2_ values [212]. It should be mentioned that beyond ~25 × 10^6^ Hz, there was a slight increase in the *ε*_2_ values. Considering the Ag doping ratio at 1 kHz, the variation in the *ε*_2_ values is displayed in Figure 12 [175].

The conclusion of this study was that the *ε*_2_ values for all Ag-doped samples were inferior to those corresponding to simple Sr apatite [175].

Helen S. and Kumar A.R. [26] explored the frequency dependence of *ε*_2_ values in samples with different concentrations. The elevated *ε*_2_ values observed at low applied frequencies were related to diverse polarizations, including space charge, ionic, dipolar, and electronic polarizations within the ion-doped HA samples [201]. In addition, the decreased *ε*_2_ values with an increase in the applied frequency indicated the existence of a dielectric relaxation within the prepared samples [26].

In a complementary study aiming to examine the dissipated energy in the prepared samples, the evolution of the *ε*_2_ values was investigated as a function of the applied frequency, across the 10 Hz–10 MHz domain, at RT. It was demonstrated that the *ε*_2_ values were higher at low applied frequencies for zero DC bias voltage, reflecting dipole oscillations. As the ionic polarization ceased at high applied frequencies, no energy was expended to rotate the ions’ dipole, resulting in decreased *ε*_2_ values [203]. A dispersion in the *ε*_2_ values was found due to varying Cu concentrations at low applied frequencies. In addition, it is important to mention that both *ε*_1_ and *ε*_2_ values exhibited similar behavior at high applied frequencies. Under a 10 V DC bias voltage, the inferred *ε*_2_ values were higher at 10 Hz and diminished when the applied frequency was increased. Thus, it was indicated that the increased polarization was due to the DC bias voltage. Up to 1 kHz, different DC bias voltages led to a distribution in the *ε_2_* values across all investigated samples. However, up to 10 MHz, this distribution markedly decreased when the applied frequency was increased [170].

A comparable decreasing trend of the *ε*_2_ values was also indicated when increasing both the applied frequency and the CuO and HT contents [115]. One possible explanation for this decrease was related to the insufficient time for the electric dipoles to align themselves with the rapidly changing direction of the applied electric field. When considering the CuO and HT higher contents, the explanation for the reduced *ε*_2_ values was connected to the increased number of charge carriers in the system [213]. Interestingly, a contrasting behavior in the *ε*_2_ values was observed after immersing the samples in SBF, as they exhibited an increase following immersion [115].

**Figure 13:**
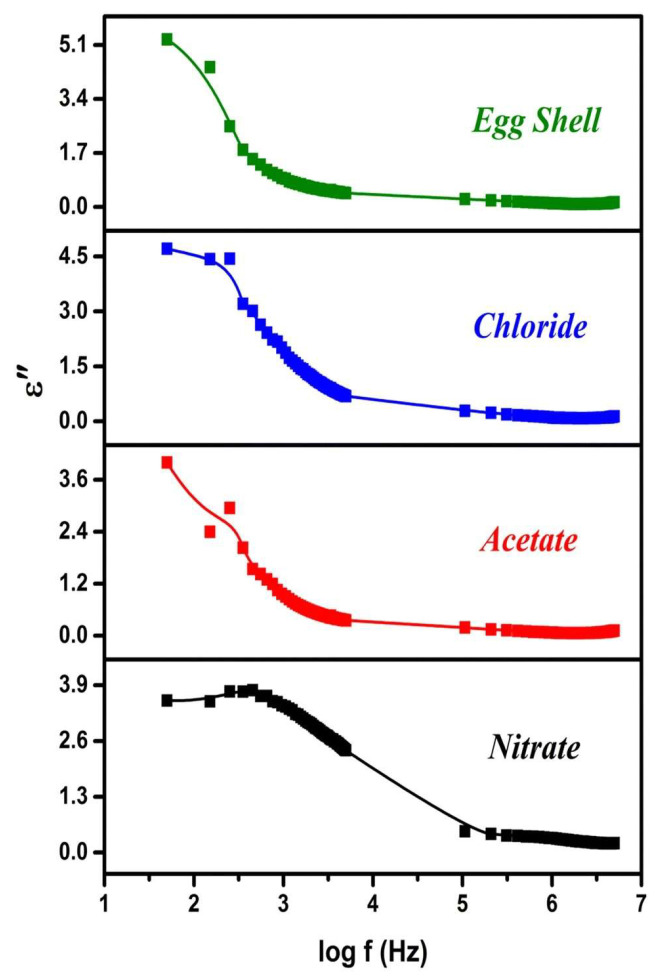
Dielectric loss (*ε*″—referred to as *ε*_2_ in the text) as a function of frequency (*f*) plots for the hydroxyapatite samples [184].

Sundarabharathi et al. [184] also observed that when increasing the applied frequency, the *ε_2_* values of HA samples decreased (Figure 13). This phenomenon was related to the ions’ movement within all the prepared samples.

Moreover, no important changes in the *ε*_2_ values were found for the doped HA samples when applying a frequency of 5 MHz [184].

##### Tangent Loss

Chatterjee et al. [85] reported that the *tan*(*δ*) values for all their prepared samples were less than 0.5. This finding indicated an irrelevant quantity of thermal energy loss that was converted into heating energy within the material.

**Figure 14:**
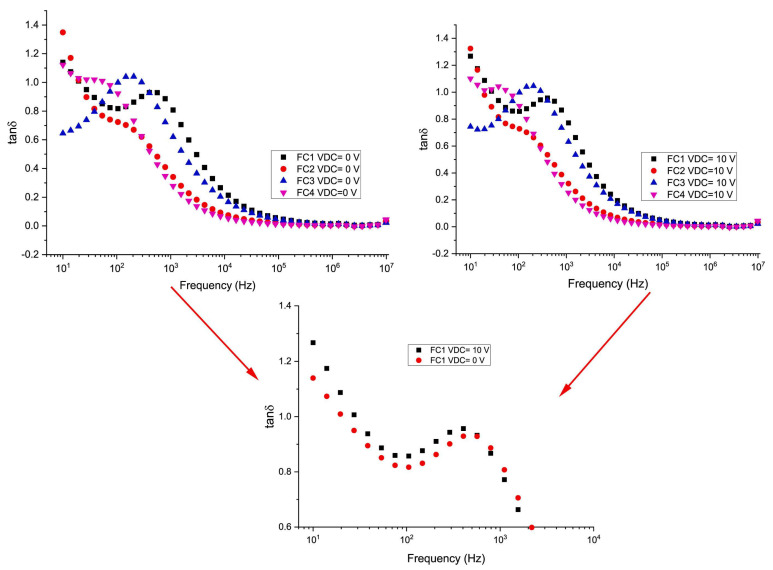
Tangent loss (*tan*(*δ*)) as a function of frequency for all the samples for the bias voltages of 0 and 10. Reproduced with permission from [170].

The behavior of the applied frequency function of the *tan*(*δ*) was evidenced by Ercan et al. [170], particularly focusing on zero DC bias voltage conditions (Figure 14).

Therefore, it was observed that the maximum loss was observed at both 200 and 500 Hz, displaying a peak that decreased as the applied frequency increased. This peak designated a resonance between the hopping motion frequency and the external AC electric field frequency. A comparable phenomenon was also observed elsewhere [20]. At very low applied frequencies (i.e., 40 and 100 Hz), a plateau was evidenced, with *tan*(*δ*) values decreasing as the applied frequency increased. This observation was related to the higher energy requirement at such low applied frequencies, causing the dipole moment of an (OH^−^) group to follow the applied AC electric field. As a consequence, higher *tan*(*δ*) values resulted. When increasing the applied frequency, the dipole moment could not keep up with the applied AC electric field, requiring lower energies and leading to decreased *tan*(*δ*) values. An interesting observation implied the shift of the maximum peak’s position toward low applied frequencies along with increasing Cu concentration. Comparative results were reported in studies involving HA structures [214]. Complementary investigations have demonstrated that the modifications in the *tan*(*δ*) for simple HA structures are related to reorientations of (OH^−^) groups [25,215,216].

The behavior of *tan*(*δ*) as a function of applied frequency under a DC bias voltage of 10 V has also been investigated (Figure 14). As compared with the zero DC bias voltage, no significant differences were observed at 10 V, except for the samples containing the lowest concentration of Cu (FC1) within the frequency range of 10 Hz to 1 kHz (Figure 14). Therefore, it could be concluded that DC bias voltage influenced polarization, especially when applying small frequencies in the case of Fe-based samples (FC1). However, this effect disappeared in all other samples with varying concentrations of Cu (i.e., FC2–FC4) [170].

#### 5.5.4. Permittivity

Yahia et al. [182] demonstrated that, at RT, the values of the relative permittivity (*ε_r_*) increased with the applied frequency, and this behavior was consistent across all investigated samples. It is important to note that the increased *ε_r_* values at low applied frequencies were reported to be related to the presence of either Maxwell Wagner relaxation or electrode and space charge polarizations [217].

**Figure 15:**
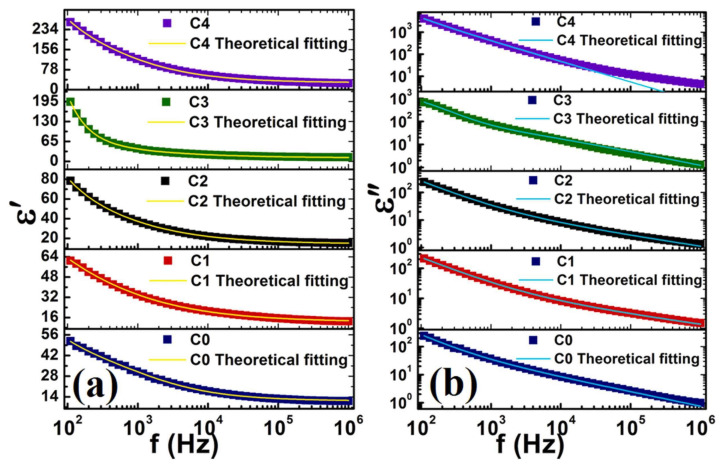
(**a**) The real and (**b**) imaginary parts of complex permittivity (ε′, ε″—referred to as *ε_r_* in the text) vs. frequency (*f*) with modified Cole–Cole fitting C0–C4 samples at 613 K. Reproduced with permission from [85].

In a complementary study conducted by Chatterjee et al. [85], the dependence of the applied frequency on both the real and imaginary parts of complex dielectric permittivity was found for undoped and Ag-doped HA samples prepared at 613 K (Figure 15a,b).

Thus, for the simple HA samples (C0), the values observed for the real permittivity ranged from 14 to 55 in the high- to low-frequency range. In contrast, samples containing the highest Ag doping concentration (C4) exhibited a progressive increase, reaching an approximate value of 230 [85].

**Figure 16:**
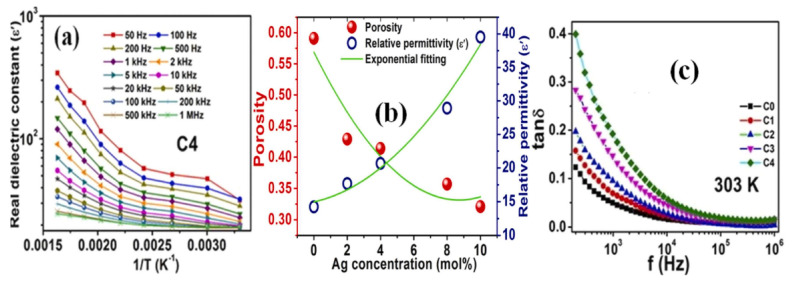
(**a**) The real part of complex permittivity (*ε*′—referred to as *ε_r_* in the text) vs. inverse temperature (1*/T*) for several frequencies, (**b**) variation of porosity (*P*) and relative permittivity (*ε*′—referred to as *ε_r_* in the text) as a function of doping concentration, and (**c**) frequency (*f*) variation of dielectric loss (*tan*(*δ*)) for all samples investigated at 303 K. Reproduced with permission from [85].

Figure 16 reveals the thermal variation of the real part of *ε_r_* for the C4 sample across various frequency values (Figure 16a), the variation of porosity and *ε_r_* depending on the doping concentration (Figure 16b), and the frequency-dependent changes in *tan*(*δ*) values for both undoped and doped HA samples (Figure 16c) [85].

Unlike the large and bulky crystallites observed in samples with a reduced content of Ag ions, those with an increased concentration of Ag exhibited smaller crystallites densely packed in a given space. Therefore, a decrease in the pore spaces could be observed, along with an increase in the bulk density for the Ag-doped HA samples. An important deterioration of the porosity was also observed, significantly impacting the dielectric permittivity as the lattice became denser [85]. The porosity values for C0–C4 samples were inferred, and a comparative plot of porosity and *ε_r_* as a function of doping concentration is presented in Figure 16b. It is important to mention that the inverse relationship between porosity and the *ε_r_* values observed at RT [32] in both undoped and Ag-doped HA samples undoubtedly evidenced the crucial role of samples’ bulk porosity in determining dielectric characteristics. The significant increase in the values of the complex permittivity with the temperature was related to the existence of a substantial polarization needed to generate the necessary surface potential [85].

### 5.6. Simple and Doped Hydroxyapatite Pellets

Table 2 introduces information related to the materials used, synthesis techniques, and tackled electrical parameters in the case of HA pellets. For more detail on the studies, refer to the corresponding references provided in the last column of Table 2.

#### 5.6.1. Impedance Spectroscopy

**Figure 17:**
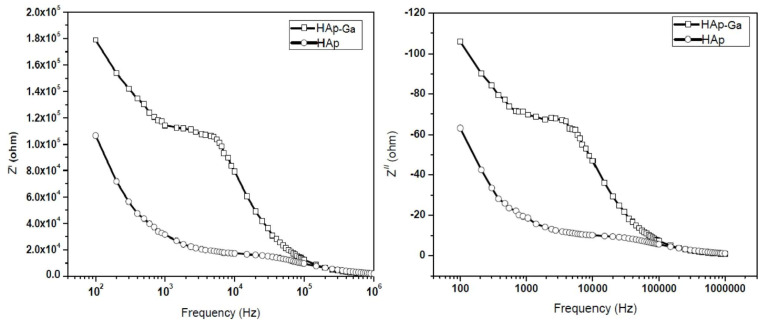
Frequency vs. real (*Z*′) and imaginary (*Z*″) parts of complex impedance of hydroxyapatite (HA) and Ga-doped HA. Reproduced with permission from [171].

Figure 17 presents the complex impedance of HA and Ga-doped HA samples across a frequency range spanning from 100 Hz to 1 MHz, at RT.

It has been observed that the real part (*Z*′) of impedance presents a steady decrease with the applied frequency. The increased *Z*′ values at lower frequencies were related to a polarization increase [221]. In the case of the imaginary part (*Z*″), its values decreased as the applied frequency increased and displayed a peak, referred to as the relaxation frequency of the HA–Ga material, in contrast to simple (undoped) HA. It was, therefore, suggested that the reorientation of dipoles and the supportive ions’ motion could disrupt the surrounding electrical potential [171]. Furthermore, both interfacial and dipolar polarizations were indicated to be influenced by the particle’s grain size. Thus, larger grains facilitated an easier electron flow towards the grain boundaries, leading to enhanced interaction between adjacent grains [171].

It is known that barrier layers are formed due to dangling bonds and nonstoichiometric oxygen present on grain boundaries. This effectively traps charge carriers. Hence, these grain boundaries exhibit a response at lower frequencies in comparison to the grains, owing to their superior resistance and capacitance values [222,223,224]. In the case of Cr-doped HA samples, the application of various heating temperatures, along with the tuning of the microstructural heterogeneities, such as grains, grain boundaries, and/or voids, determined the appearance of multiple semicircular arcs in the impedance plane plots. This occurrence was related to the distinct mean relaxation frequencies (f_r_ = 1/2πRC) of the charge carriers. One should note that the lowest electrical resistance values were inferred in the case of undoped HA samples, in comparison to Cr-doped ones. It was also observed that an increase in the heating temperature up to 200 °C resulted in elevated electrical resistance values. This result could be connected to a collective effect of bigger Cr (III) valance states, the addition of oxygen during heat treatments, the loss of surface adsorbed water, and the disruption of intimate contact between intrinsic grains. Another significant finding was the presence of at least two semicircles, small and large, when applying higher and lower frequencies [169]. When compared with undoped HA, the resistances of each component initially decreased, followed by an increase with the addition of Cr. This was related to a lower number of voids [225]. At 200 °C, this number increased, leading to reduced contact area of the grain boundary and the formation of open pore structures around the grain boundaries. Consequently, an increase in resistance values was observed [169].

The impedance formalism, represented by *Z*″ as a function of the applied frequency elucidated the highly resistive phase. For the case of both simple and Cr-doped (at 100 °C) HA samples, no relaxation peaks could be observed at high frequencies. However, incomplete relaxation peaks appeared at low applied frequencies. This behavior was attributed to voids and grain boundaries, in which ionic species exhibited their relaxation below 1 Hz. When increasing the temperature to 200 °C, and at an applied frequency of 10^3^ Hz, the investigated samples evidenced a broad peak. This phenomenon was connected to the close relaxation frequency associated with charged species at both conventional and unconventional grain boundaries [169].

When examining the modulus (M″) formalism, in which M″ is plotted as a function of the applied frequency, the material’s bulk properties can be deduced. Thus, when plotting M″ vs. log(f) for both simple and Cr-doped (at 100 °C) HA samples, incomplete broad relaxation peaks at high applied frequencies (i.e., >10^6^ Hz) were evidenced. The occurrence of these peaks was connected to the short-range mobility of charge carriers, which contributes to the capacitance originating from the intrinsic grains [226].

The results of the impedance spectra reported by Nisar et al. [218] demonstrated that the values of the total HA resistance decreased with the Ce doping concentration.

It is believed that pure HA is a weak conductor [227], and the contribution of both Ca^2+^ and PO_4_^2−^ ions to conductivity is negligible. The exact conductivity mechanism of HA remains unclear, but it has been proposed that OH^−^ ions, protons (H^+^), or holes could serve as the primary charge carriers. It is thought that OH^−^ ions migrate toward the center of the Ca^2+^ triangles along the *c*-axis. Additionally, protons may hop along with neighboring (OH^−^) [228], PO_4_^3−^ or O^2−^ ions. The larger distance between two adjacent OH^−^ ions (0.344 nm) compared with PO_4_^3−^ ions (0.307 nm) makes proton interaction with neighboring PO_4_^3−^ ions more favorable. This increase in conductivity holds potential for applications in bone healing and repair [229].

One should note that, in general, a higher impedance modulus at lower frequencies suggests a greater corrosion resistance of the investigated samples [139].

It has been shown that, at lower frequencies, impedance values increase due to Maxwell–Wagner polarization [230]. As the frequency increased, the impedance values decreased and merged due to the halting effect of space charge polarization. With increasing Zn^2+^ ion concentration, the impedance values decreased, which led to an increase in conductivity. This is indicative of higher space charge ion concentration [231]. Consequently, the ZnHA impedance values gradually decreased from 4 to 1 wt.%. Initially, at an applied frequency of 100 Hz, the impedance values corresponding with undoped HA were inferior to those observed in the case of doped samples. It is important to mention that the impedance values increased with the Zn concentration. However, as the values of the applied frequency increased, the impedance values decreased and stabilized [177].

In another study, it was reported that as the concentration of Mg^2+^ ions increased, replacing Ca^2+^ sites, the impedance values decreased. The impedance values were also shown to decrease with the applied frequency. This trend of decreasing impedance values with increasing frequency is inversely proportional to conductivity. Consequently, the decrease in impedance values observed in the case of all investigated AgHA samples implied improved conductivity [179].

#### 5.6.2. AC Conductivity Analysis

**Figure 18:**
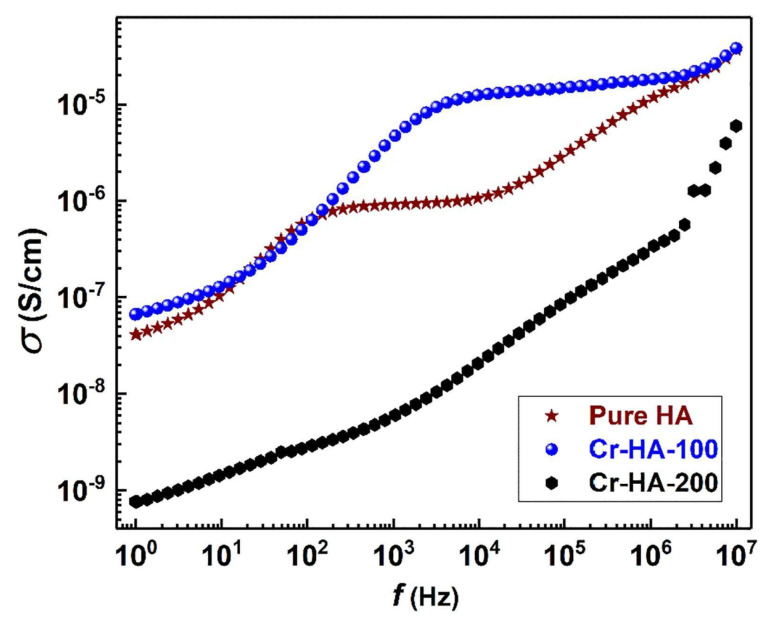
Log–log plot of AC conductivity (*σ_ac_*) vs. frequency (*f*) for undoped HA, Cr-doped hydroxyapatite treated at 100 °C (Cr–HA-100), and Cr-doped hydroxyapatite treated at 200 °C (Cr–HA-200) samples. Reproduced with permission from [169].

Iqbal et al. [169] reported that the electrical conductivity values corresponding to pristine and Cr-doped HA (100 °C) samples exhibited two distinct dispersion regions (Figure 18).

When increasing the temperature (i.e., 200 °C), it was observed that the Cr-doped HA sample lacked a clear dispersion region. Compared with undoped HA, the Cr-doped HA (100 °C) samples displayed higher conductivity in the low-frequency region, merging with undoped HA’s conductivity at approximately 10^6^ Hz. This increase in HA conductivity due to Cr doping suggested an improvement in its bio-conductivity, which confirmed the successful incorporation of Cr ions into the HA structure [53]. In their study, Iqbal et al. [169] evidenced that the effective conductivity of undoped HA exhibited a tendency to increase with the applied frequency, displaying a dispersive behavior at low applied frequencies. This behavior could be attributed to the hopping of ions, such as H^+^, between localized O^2−^ ions or their interaction with the double-bonded oxygen of the PO_4_^3−^ group [232]. At low frequencies, the enhanced ionic conductivity of Cr-doped HA (100 °C) samples, compared with undoped HA ones, was attributed to the presence of multivalent Cr ions (both Cr (III) and Cr (IV) ones) at the OH^−^ site, along with H^+^ ions. However, at higher temperatures (i.e., 200 °C), the conductivity of the Cr-doped HA samples decreased. Previous studies have linked RT conduction in HA to the migration of H^+^ ions in adsorbed water, suggesting that OH^−^ ions could promote and impede H^+^ ion conduction at high temperatures [233]. Thus, in these specific Cr-doped HA samples, the higher concentration of Cr (III) ions replaces H^+^ ones at OH^−^ sites, thus hindering H^+^ ion conduction and reducing overall conductivity [234,235]. The investigation of electrical conductivity revealed that the atomic ratio of Cr (III)/Cr (IV) ions is influenced by the final heating temperature, thereby regulating the sample’s conductivity. It is important to note that the atomic composition of Cr (IV) ions had a minimal impact and its value remained unchanged, even at elevated sintering temperatures [169].

Nayak B. and Misra P.K. [171] demonstrated in their work that, within the frequency range of 10^2^–10^3^ Hz, the *σ_ac_* values remained nearly constant for all investigated samples. Upon further frequency increase (starting from 10^5^ Hz), the *σ_ac_* values showed a linear increment. In the low-frequency region, the gradual rise in value was attributed to the static movement of ions carrying charges. Due to the weak electric field, charge accumulation at the interface was minimal [236]. With the increase in the field strength, ions migrate swiftly to the materials’ interface, leading to enhanced *σ_ac_* values [237]. The conductive nature of HA is usually connected to the hopping movement of surface protons among its ionically active sites (O_2_ anion and OH^−^ ion) along the *c*-axis [238]. Because the *σ_ac_* measurement was conducted at RT, ion migration in adsorbed and/or condensed water is believed to contribute to surface conduction in HA [78]. Interestingly, as compared with undoped HA, the introduction of Ga ions into the HA structure did not significantly alter the *σ_ac_* values. This suggests that Ga ions do not play a prominent role in the material’s dielectric behavior. At elevated frequencies, rapid forward–backward hopping facilitates the long-range diffusion of ions [239,240]. The interaction of Ga^3+^ with HA has been shown to disrupt the vibration of surface (OH^−^) groups, potentially disordering the apatite crystal’s interface upon Ga^3+^ ions incorporation [46]. However, the interaction of electrically active O_2_ anions with the double-bonded oxygen PO_4_ groups remained unaffected. Additionally, the substitution of Ga^3+^ ions with Ca^2+^ ions through ion exchange effectively enhanced the conductivity values because Ga itself was demonstrated as an excellent ionic conductor [171].

**Figure 19:**
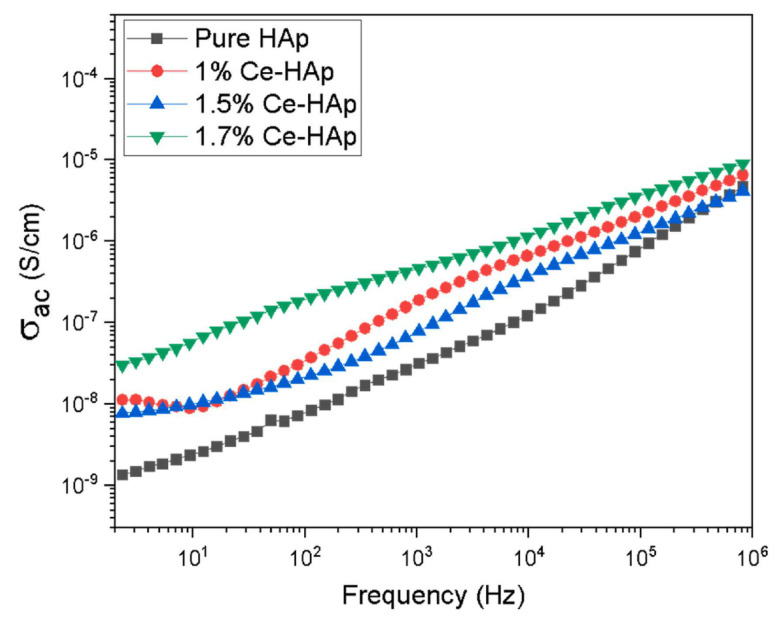
Relationship between AC conductivity (*σ_ac_*) and frequency for undoped and Ce-doped green hydroxyapatite samples. Reproduced with permission from [218].

Nisar et al. [218] reported on the correlation between *σ_ac_* and applied frequency (Figure 19).

Thus, it was demonstrated that the *σ_ac_* values corresponding to Ce-doped HA, with a maximum of 1.7% Ce content, were superior to those corresponding to undoped HA. This enhanced conductivity could be attributed to the higher charge density of Ce compared to Ca^2+^, which is compensated by an increased number of charge carriers, such as protons and/or holes. The in-situ replacement of Ca^2+^ ions with Ce ions resulted in a uniform distribution of doped particles within the HA structure. This uniform distribution likely improved the electrical network formation, consequently enhancing the conductivity of the doped samples beyond that corresponding to undoped HA. It was, therefore, demonstrated that the effective conductivity of HA has a tendency to increase with the applied frequency. The low-frequency dispersive behavior was connected to the hopping movement of H^+^ ions with O^2−^ ions or their interaction with the doubly bonded oxygen of (PO_4_)^3−^ groups [232]. At low applied frequencies, the increase in conductivity with higher doping percentages is linked to the presence of multivalent Ce ions at the OH^−^ sites, in addition to H^+^ ions. In ceramics, ions are considered network channels of conductive (R) and capacitive (C) sites. In the case of HA, columnar OH^−^ or H^+^ ions could be regarded as conductive sites, whereas immobile ions could serve as capacitive sites [218].

The behavior of *σ_ac_* in all investigated samples was evidenced by Ercan et al. [101], covering frequencies from 10 Hz to 10 MHz. The frequency-independent portion of *σ_ac_*, which remains constant up to 10^5^ Hz, was related to DC conductivity. Subsequently, *σ_ac_* showed a rapid increase with escalating frequency. The inferred frequency-dependent *s* values obtained from the slopes of the linear segments exhibited a distribution that changed below unity (i.e., 0.7114 ± 0.02323, 0.72388 ± 0.02267, 0.80347 ± 0.01478, 0.68179 ± 0.02501, and 0.69302 ± 0.02321 for HA, 0.37Sr–HA, 0.37Ni–0.37Sr–HA, 0.74Ni–0.37Sr–HA, and 1.11Ni–0.37Sr–HA, respectively) [101]. It is important to observe that the addition of Sr to simple HA led to an increase in the *s* values. In Sr-doped HA samples, as the Ni content increased, the *s* values initially raised and then decreased. Thus, it could be concluded that there was no measurable direct current conductivity. This finding aligned well with the frequency-independent AC conductance detected up to 10^5^ Hz [101]. It should also be emphasized that, at high applied frequencies, the *σ_ac_* values of the investigated samples indicated sudden hopping with translational motion [194,202,241,242].

#### 5.6.3. Dielectric Studies

Exploring the electrical properties of bone and HA is essential due to bone’s composition, which includes HA as a mineral component. Bone comprises intricate pores filled with fluid and collagen, which makes this structure more complex than that corresponding to HA [177].

##### Dielectric Constant

**Figure 20:**
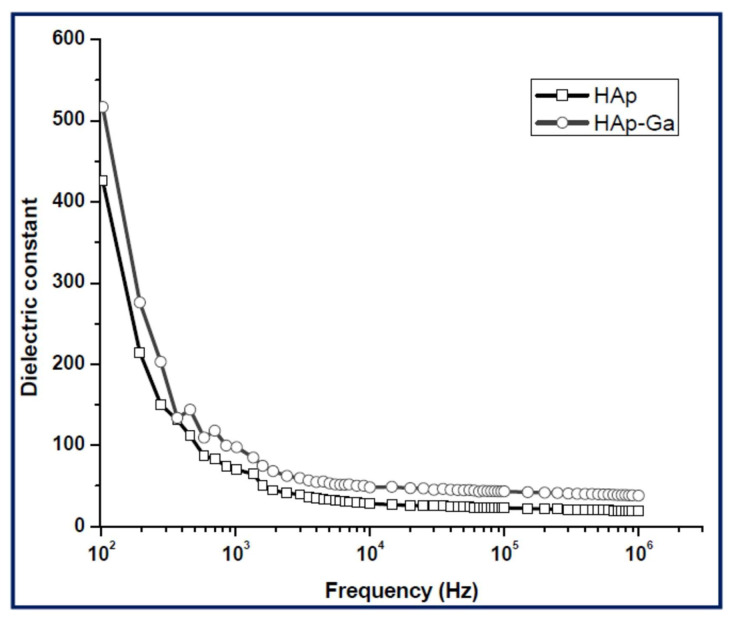
Frequency vs. dielectric constant plot. Reproduced with permission from [171].

It is known that the *ε*_1_ is a fundamental parameter that characterizes the polarizability of a dielectric material under applied alternating current [171]. The typical decrease in the *ε*_1_ values with increasing the applied frequency was observed by Nayak B. and Misra P.K. in both their investigated samples (i.e., undoped and Ga-doped HA) (Figure 20).

From the results presented in Figure 20 it can be seen that the maximum *ε*_1_ values, i.e., 426 for undoped HA and 516 for Ga-doped HA, were observed at a frequency value of 10^2^ Hz. The elevated *ε*_1_ value was correlated with the strong electric polarization in the Ga-doped HA bioceramics. At higher frequencies, which correspond to interfacial polarization, the *ε*_1_ ultimately decreased to lower values [171,243].

The introduction of Mg ions was also shown to enhance the *ε*_1_ values [2]. At low applied frequencies, the high *ε*_1_ values were correlated with the ionic polarization, but it subsequently decreased due to the lag in dipole response to the electric field [244]. The incorporated samples showed enhanced *ε*_1_ values at lower applied frequencies, possibly due to the increased ionic polarization [2].

The obtained *ε*_1_ values for both undoped and Cr-doped HA (at 100 °C) samples, especially in the low (10^2^ Hz) and intermediate (10^5^ Hz) frequency ranges, surpass those reported for ceramic materials, thin films, and nano-powders of both undoped and doped HA materials [10,245,246,247]. However, in the high-frequency regime (1–5 MHz), the observed *ε*_1_ values are comparable to the reported values. In the low-frequency range, the relatively enhanced *ε*_1_ values were due to improved interfacial polarization for undoped HA and additional dipoles formed from Cr (III) and Cr (IV) ions in Cr-doped HA (100 °C) sample. It was concluded that the superior electrical conductivity and high dielectric constant with lower dielectric loss of the Cr-doped HA (100 °C) samples proved their potential to be used as excellent dielectric materials for applications in bone growth, repair, and regeneration [169].

**Figure 21:**
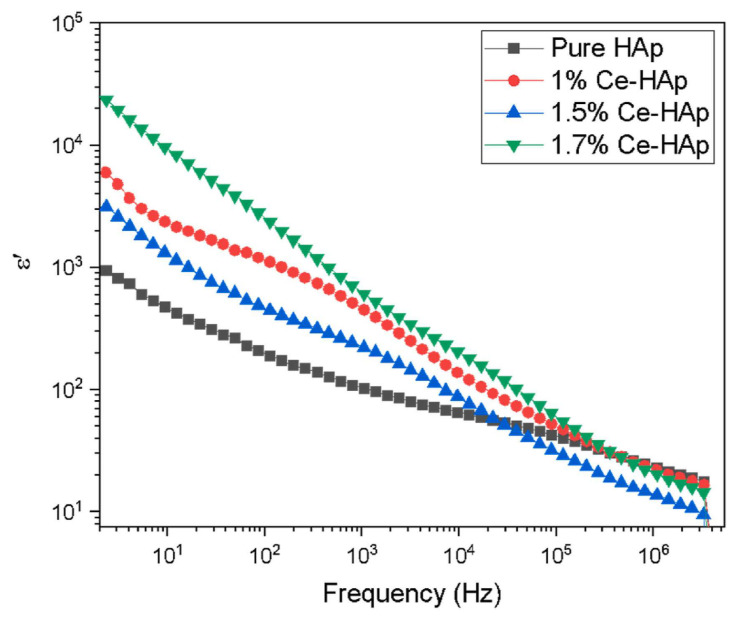
Relationship between the real part of dielectric constant (*ε*′—referred to as *ε*_1_ in the text) and frequency for undoped and Ce-doped green hydroxyapatite samples. Reproduced with permission from [218].

In a comparative study, changes in the real part of *ε*_1_ with frequency were reported (Figure 21) [218].

Thus, it was demonstrated that the *ε*_1_ real part exhibited a sharp decrease while increasing the applied frequency. In the low-frequency range, it was observed that the *ε*_1_ values were elevated, but they decreased with an increase in the applied frequency. This behavior was likely due to the electric dipoles being unable to respond quickly to the changing AC field. Interestingly, the inferred *ε*_1_ values were superior for Ce-doped HA samples in comparison to undoped HA ones. This result was anticipated due to the enhanced *σ_ac_* values corresponding with Ce-doped HA samples. Upon Ce doping, the *ε*_1_ real part was lowest for the 1.5% substitution concentration, and a slight increase was observed for the 1.7% substitution concentration due to a phase change. Therefore, it could be concluded that a concentration of 1.5% Ce in HA might be suitable for biomedical applications, offering higher *ε*_1_ values in comparison to undoped HA [218].

It is well-known that the HA structure comprises ionic species such as Ca^2+^, PO_4_^3−^, and OH^−^, which form a channel along the *c*-axis. The dipole moment of OH^−^ ions significantly contributes to *ε*_1_, alongside minor contributions from ionic species, given that HA is an ionic ceramic. In the low-frequency range, the *ε*_1_ values are higher because the frequency of the applied electric field matches the natural frequency of the bound charge (OH^−^), causing molecular oscillation. Both electronic and ionic polarization mechanisms are active in this region, enhancing the *ε*_1_ values. Though, at higher frequencies, the ionic dipole moment struggles to align with the applied field, which results in a lag in their direction and a reduction of the *ε*_1_ values [248]. For 1.5% Ce-doped HA, the decrease in the *ε*_1_ and *σ_ac_* values, along with the increase in resistance, could be attributed to the development of a secondary phase (Ca_3_Ce(PO_4_)^3−^), which indicated by XRD analysis. At this composition, the secondary phase began to emerge but was not fully formed. The presence of competing phases may lead to diverse behaviors. As the second phase fully developed in 1.7% Ce-doped HA, the *ε*_1_ and *σ_ac_* values started to increase, accompanied by a corresponding decrease [218].

**Figure 22:**
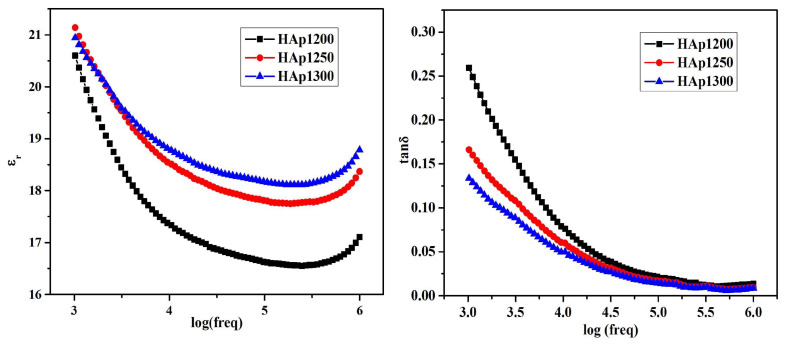
Variation in *ε_r_* (referred to as *ε*_1_ in the text) and *tan*(*δ*) with frequency at room-temperature sintered hydroxyapatite samples [219].

The relationship between *ε*_1_ and *tan*(*δ*) at RT across frequencies ranging from 1 kHz to 1 MHz for HA samples sintered at various temperatures is illustrated in Figure 22 [219].

A consistent trend of decreasing *ε*_1_ with increasing frequency was observed in all sintered samples. This decline was related to a reduction in net polarization. Higher *ε*_1_ values at low frequencies were linked to enhanced space charge polarization within the investigated samples [219]. At RT, it was noted that an increase in the sintering temperature led to a rise in the *ε*_1_ values. This enhancement in *ε*_1_ with higher sintering temperatures was likely connected to the density and the grain size of the sintered HA samples [249]. In their work, Swain et al. [219] demonstrated that the *ε*_1_ values corresponding with HA samples sintered at both 1300 and 1250 °C were nearly identical, the highest one being approximatively 21 at 1 kHz, at RT. The variation in *ε*_1_ at different frequencies (ranging from 1 kHz to 1 MHz) with the temperature of the sintered HA samples was also shown. A noticeable dielectric anomaly, shifting toward elevated temperatures as the applied frequency increased, was evidenced in all the sintered samples. Specifically, in all sintered HA samples this anomaly occurred around 130 °C, 175 °C, 210 °C, and 285 °C at frequencies of 1 kHz, 10 kHz, 100 kHz, and 1 MHz, respectively. Notably, a dielectric anomaly was also detected around 210 °C. This phenomenon was previously reported in the literature [250] and associated with the re-orientational motion of OH^−^ ion dipoles [219].

**Figure 23:**
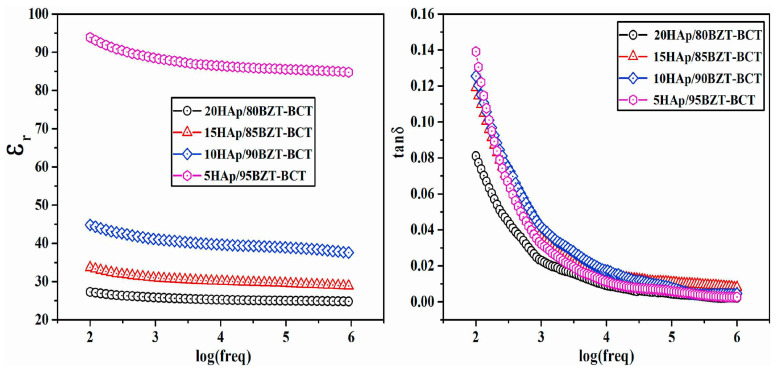
Room-temperature variation of dielectric constant (*ε_r_*—referred to as *ε*_1_ in the text) and dielectric loss (*tan*(*δ*)) with frequency of hydroxyapatite/(0.5Ba(Zr_0.2_Ti_0.8_)O_3_-0.5(Ba_0.7_Ca_0.3_)TiO_3_) (HA/BZT–BCT) composites. Reproduced with permission from [59].

The RT variation of *ε*_1_ with frequency (ranging from 100 Hz to 1 MHz) is plotted in Figure 23 [59].

Therefore, it was found that the *ε*_1_ values corresponding to all investigated composite samples decreased with the increase in frequency, which was most likely due to a net decrease in polarization [17]. Additionally, it was observed that the *ε*_1_ values increased with the rise in BZT–BCT wt.% in BZT–BCT/HA composite samples. The highest value of *ε*_1_ observed at RT (i.e., ~94 at an applied frequency of 100 Hz) was indicated in the case of 5% HA/95%BZT–BCT composite samples. The modification with HA, having a lower *ε*_1_ value, of BZT–BCT, having a higher *ε*_1_ value, resulted in a significant decrease in the *ε*_1_ value of composites in comparison to a simple BZT–BCT system [251]. It is known that the *ε*_1_ value of a system is influenced by its microstructure, density, presence of intermediate phases, porosity, and grain size distribution. A notable difference in the *ε*_1_ values was observed between the 5% HA/95% BZT–BCT composite and the other investigated samples. This reduction in the *ε*_1_ values in composites with higher percentage of HA can be attributed to the formation of intermediate phases such as CaZr_4_O_9_ and Ba_3_(PO_4_), which have relatively lower *ε*_1_ values [252,253]. It is well-known that the reported *ε*_1_ values for the human bone falls in the range of 8–10 [254]. It is important to mention that, in the aforementioned study, enhanced *ε*_1_ values were obtained in the case of composite samples in comparison with undoped HA [219].

**Figure 24:**
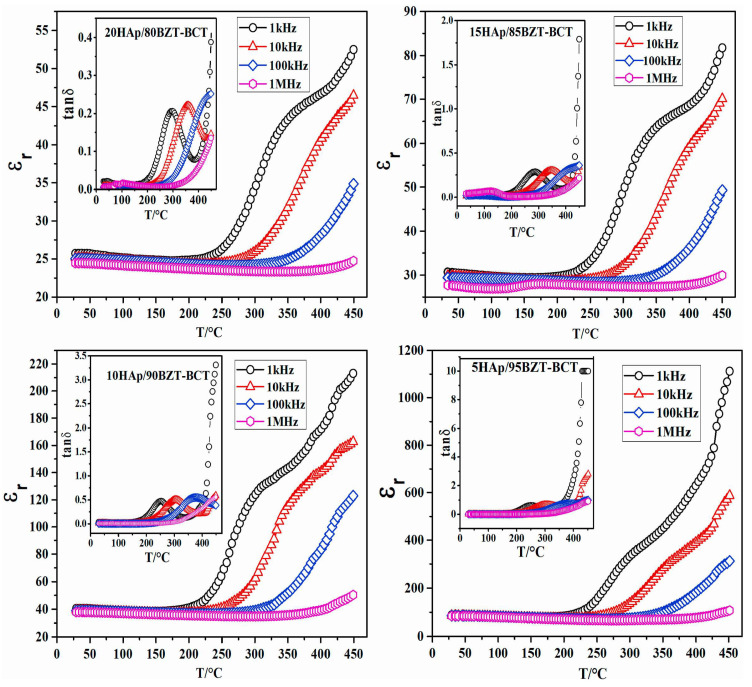
Temperature dependence of dielectric constant (*ε_r_*—referred to as *ε*_1_ in the text) and dielectric loss (*tan*(*δ*)) of hydroxyapatite/(0.5Ba(Zr_0.2_Ti_0.8_)O_3_-0.5(Ba_0.7_Ca_0.3_)TiO_3_) (HA/BZT–BCT) composites. Reproduced with permission from [59].

The variation of *ε*_1_ at different frequencies with the temperature of HA/BZT–BCT composite samples is illustrated in Figure 24 [59].

Therefore, it was demonstrated that the *ε*_1_ values increased with the applied temperature, reaching a plateau region from RT to 200 °C. As shown, the dielectric anomalies at 1 kHz and 10 kHz frequencies occurred at around 350 °C and 400 °C for both 20% HA/80% BZT–BCT and 15% HA/85% BZT–BCT composite samples, respectively. In contrast, 10% HA/90% BZT–BCT and 5% HA/95% BZT–BCT composite samples exhibited dielectric anomalies at lower temperatures, approximatively 300 °C and 375 °C. This discrepancy could be attributed to the formation of smaller-sized grains in 10% HA/90% BZT–BCT and 5% HA/95% BZT–BCT composites compared with 20% HA/80% BZT–BCT and 15% HA/85% BZT–BCT composites [255]. The Curie temperatures (T_c_) pertaining to HA and BZT–BCT systems are reported to be ~210 °C and ~94 °C, respectively [219,251]. Broad peaks indicating dielectric anomalies were observed in all the investigated composites, shifting to higher temperatures with increasing applied frequencies. This behavior is characteristic of relaxor ferroelectrics and is associated with diffuse phase transition behavior [256]. It was demonstrated that the temperature dependence of *ε*_1_ in composites is influenced by the dielectric behavior of HA, BZT–BCT, and intermediate phases present in the composite materials. With an increase in BZT–BCT wt.% in the composites, *ε*_1_ values raised, with the highest observed value found in the case of 5% HA/95% BZT–BCT composite samples [59].

The *ε*_1_ values for HA–BTS sintered composites were also measured by Uribe et al. [220] in the frequency range of 100 Hz to 100 kHz. Thus, it was shown that the *ε*_1_ values decreased when increasing the applied frequency. Notably, at each frequency, the *ε*_1_ values significantly decreased with the increase in the HA content: at 100 Hz, the *ε*_1_ values corresponding to 10% HA–90% BTS composite samples decreased by ~40% with a 10% addition of HA as compared with the *ε*_1_ values of simple BTS samples. Several factors were indicated to contribute to the reduction in the effective *ε*_1_ values: grain structure, lack of connectivity between phases, formation of sintered reaction products, and porosity. It is worth mentioning that the piezoelectric coefficient for human bone is reported to be 0.7 pC/N [257]. Interestingly, the compound 70% HA–30% BTS exhibited a piezoelectric coefficient of 2.6 pC/N, which indicates a significant improvement in the piezoelectric properties upon adding BTS. This finding demonstrated that the incorporation of BTS significantly enhanced the piezoelectric properties of the composite materials.

**Figure 25:**
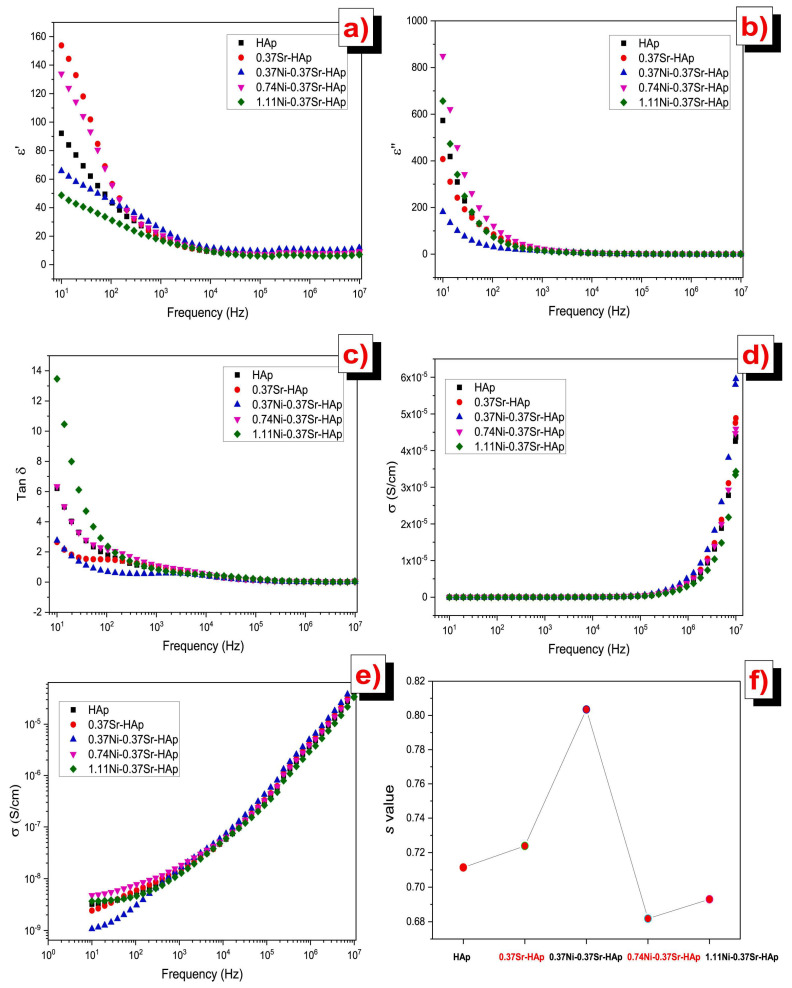
The plots of (**a**) dielectric constant (*ε*′— referred to as *ε*_1_ in the text), (**b**) dielectric loss (ε″—referred to as *ε*_2_ in the text), (**c**) loss tangent (*tan*(*δ*)), (**d**) *σac*, and (**e**) *σac* (logarithmic plot) as a function of increasing frequency and (**f**) *s* value dependent on the composition plot. Reproduced with permission from [101].

Ercan et al. [101] plotted *ε*_1_ (Figure 25a), *ε*_2_ (Figure 25b), *tan*(*δ*) (Figure 25c), *σ_ac_* (Figure 25d), and *σ_ac_* (logarithmic plot—Figure 25e) as a function of increasing frequency, and the *s* values dependent on the composition (Figure 25f). The *ε*_1_ measurements of undoped HA, Sr-doped and Sr–Ni-doped HA were conducted in the frequency range of 10 Hz and 10 MHz [101].

As can be seen in Figure 25a, the observed *ε*_1_ values were demonstrated to be dependent on the applied frequencies up to 1 kHz and independent beyond 10 kHz. The undoped HA samples exhibited specific *ε*_1_ values due to polarization. With the addition of Sr (i.e., 0.37Sr–HA), polarization increased, leading to an increase in the *ε*_1_ values. However, in Sr–Ni-doped HA samples, both the dielectric coefficient and polarization decreased with higher Ni doping levels. This change in the *ε*_1_ values can be attributed to ionic polarization. At low applied frequencies, the dipole moment of (OH^−^) groups followed the alternating external field. As the applied frequency increased, the dipole moment of (OH^−^) ions could not keep up with the external electric field. This led to a decrease in polarization and, subsequently, a decrease in the *ε*_1_ values with increasing the applied frequency [198,202,203]. Changes in additive concentration caused corresponding increases or decreases in polarization. This high polarization and consequently high dielectric coefficient property are crucial for enhanced results in bone regeneration, repair, and growth [247].

**Figure 26:**
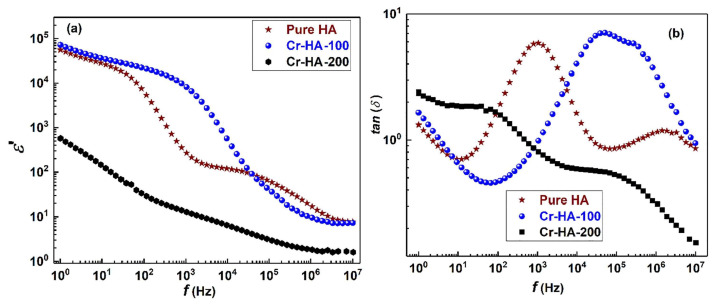
(**a**) Dielectric constant (*ε*′—referred to as *ε*_1_ in the text) and (**b**) *tan*(*δ*) vs. frequency, plotted on log scales for undoped hydroxyapatite, Cr-doped hydroxyapatite treated at 100 °C (Cr–HA-100), and Cr-doped hydroxyapatite treated at 200 °C (Cr–HA-200). Reproduced with permission from [169].

In the measured frequency range, undoped HA and Cr-doped HA (100 °C) samples exhibited one and two dispersions in their *ε_r_* response, respectively (Figure 26a) [169]. Figure 26b illustrates the variations in *tan*(*δ*) with frequency for the investigated samples. The peak observed in the *tan*(*δ*) signifies the maximum absorption of electrical energy, resulting from the interaction between the applied field and the alternating dipoles [169].

At low applied frequencies, it was demonstrated that the *ε_r_* remained relatively stable (plateau region) and then suddenly decreased at a specific frequency (ω_r_ = 2πf_r_ = 1/τ_r_). In their study, Iqbal et al. [169] plotted a log *ε_r_* function of log(f) in the case of undoped HA samples and revealed two distinct dispersions at low (~10^3^ Hz) and high applied frequencies (>10^6^ Hz), denoted as *ε_r_*_LF and *ε_r_*_HF, respectively. The *ε_r_*_LF dispersion was attributed to dominant protonic (H^+^) conduction, as protons migrate along the *c*-axis. Conversely, the *ε_r_*_HF dispersion results from the reorientation of numerous electrical dipoles of OH^−^ ions within the Ca tunnels, with the movements of these ions being sensitive to local structures and configurations [246,258].

Additionally, the higher values of *ε_r_*_LF compared with *ε_r_*_HF could be connected to interfacial polarization caused by the abnormal accumulation of H^+^ ions near grain boundaries [223,259,260,261]. A slight increase in the *ε_r_*_LF magnitude for Cr-doped HA (100 °C) samples compared with undoped HA and HA-200 samples might result from the alignment of additional dipoles associated with Cr (III) and Cr (IV) ions present at grain boundaries [199]. Interfacial polarization, arising from charge buildup at the layer-structured interface has been identified as a factor that enhances dielectric energy storage in composite materials [262,263,264]. At frequencies > 10^6^ Hz, the similar dielectric response of both undoped HA and Cr-doped (100 °C) HA could be attributed to intrinsic effects, such as local reorientation of numerous (OH^−^) dipoles in response to the applied frequency, occurring within the grain interior. In the Cr-doped HA (200 °C) samples, the overall decrease in dielectric constant at both lower and higher applied frequencies could be linked to the introduction of voids, hindering ion accumulation at grain boundaries (i.e., unconventional grain boundary regions). Furthermore, the dominance of Cr (III) ion concentrations immobilized (OH^−^) and (H^+^) related dipoles. Consequently, the *ε_r_* values were lowered, which was in line with a decrease in conductivity observed for these specific samples [169].

##### Dielectric Loss

Recently, it has been demonstrated that *ε*_2_ values exhibit frequency dependence and alter with the Ag^+^ doping. There is a consistent decrease observed with an increase in the applied frequency (ranging from 2.5 × 10^4^ Hz for 2 wt.% Ag and 3 wt.% AgHA), reaching a relatively constant value for Ag-doped (1 wt.% and 4 wt.%) HA samples up to 10^7^ Hz [175]. The *ε*_2_ values diminished as the Ag content increased due to the strong dielectric polarizing effect of Ag [265]. It was indicated that the Ag-doped (4 wt.%) and Mg-doped (3 wt.%) HA samples exhibited the lowest *ε*_2_ values. Notably, the corrosion rate and impedance values in the case of Ag-doped (3 wt.%) HA samples were also minimized, which could be connected with pore and crystallite sizes [179].

Under identical experimental conditions and applied frequency, the *ε*_2_ values observed in the case of Ga-doped HA at 10^2^ Hz decreased to 1.87 as compared with 2.3 (which corresponds to undoped HA). This result is indicative of the superior efficiency of Ga-doped HA samples as dielectric materials. It should be mentioned that this dissipation of electrical energy quantitatively occurs through various physical processes. These include electrical conduction, dielectric resonance, and relaxation, as well as losses from nonlinear processes [266].

It was also reported that in the undoped HA samples, distinct *ε*_2_ peaks, *tan*(*δ*)_LF and *tan*(*δ*)_HF, were observed at low- (~10^3^ Hz) and high-applied frequency (>10^6^ Hz) regions, respectively. These peaks corresponded to the relaxation process of dipoles at conventional grain boundaries and interiors, respectively. This observation aligns with the clear dispersions, *ε*_1__LF and *ε*_1__HF, when representing the dielectric constant function of the applied frequency. However, in the Cr-doped HA (100 °C) samples, the two dispersions were not well-resolved due to the very close relaxation times of Cr and other ions at grain interiors and conventional grain boundaries. This similarity in relaxation times was reflected in the small difference in resistance and capacitance values between intrinsic grains and conventional grain boundaries. In the case of the Cr-doped HA (200 °C) samples, the existence of two broad peaks at low and high applied frequencies could be associated with the distributed relaxation times of multivalent Cr and OH^−^ ions. This correlation extends to both conventional and unconventional grain boundaries, as well as within grain interiors [169].

**Figure 27:**
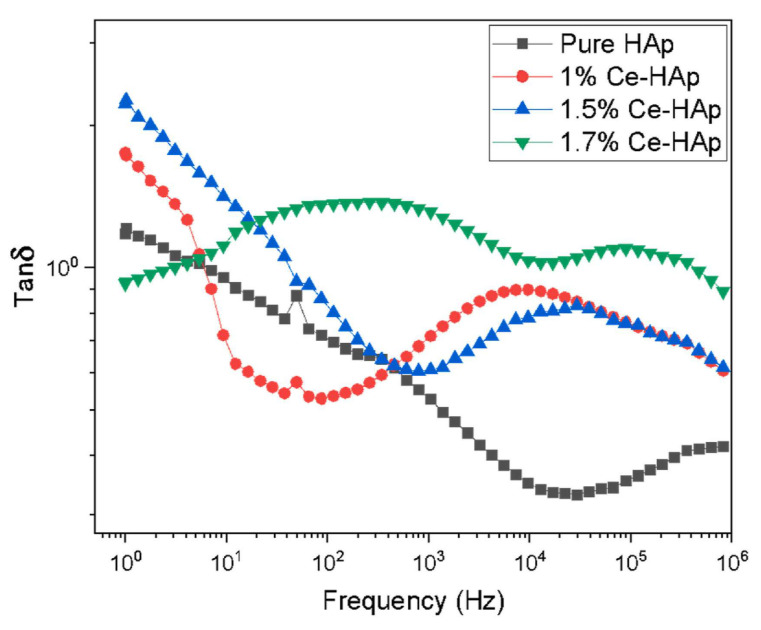
Relationship between loss tangent *tan*(*δ*) and frequency of undoped and Ce-doped green hydroxyapatite samples. Reproduced with permission from [218].

In their study, Nisar et al. [218] evidenced the variation of *ε*_2_ with applied frequency (Figure 27).

It has been observed that *ε*_2_ values are dependent on Ce dopant concentration and applied frequency. In dielectric materials, *ε*_2_ arises due to the absorption of electrical energy, which is utilized in rotating dipolar molecules. In nanomaterials, the presence of inhomogeneities can lead to the absorption of electrical energy, which results in dielectric loss. Additionally, the existence of dangling bonds on the surface may absorb gasses, which contributes to an increase in the *ε*_2_ values [267]. It has been evidenced that, at low applied frequencies, the observed *ε*_2_ values are significantly higher and decrease as the applied frequency increases, for both undoped and doped HA samples. This behavior could be attributed to the oscillations of dipoles. At higher applied frequencies, ionic polarization ceases, and energy is no longer required to rotate the dipoles. This, consequently, leads to a reduction in the *ε*_2_ values. The higher *ε*_2_ values observed in increased Ce concentrations were likely due to metal doping in HA, leading to the formation of conducting channels and facilitating leakage currents, in comparison with undoped HA samples.

Mahanty A. and Shikha D. have demonstrated, in a complementary study, the relationship between *ε*_2_ values and applied frequencies for various Mg-doping concentrations in HA [179]. Thus, the *ε*_2_ values for Mg-doped (3 wt.%) HA samples exhibited the smallest increase with the rise in the applied frequency, followed by 2, 1, and 4 wt.% Mg-doped HA. It was also reported that, at a lower frequency of 10^4^ Hz, the Mg-doped (2 wt.%) HA sample displayed the highest *ε*_2_ values. This result was connected with the dipole oscillations causing a significant increase in *ε*_2_ values at low applied frequencies. Ion polarization ceased at higher frequencies, resulting in an Mg-doped (3 wt.%) HA sample exhibiting the lowest *ε*_2_ values. Consequently, the ion dipole in HA did not rotate under the applied energy, leading to a decrease in *ε*_2_ values.

It has been indicated that the *ε*_2_ values are higher when applying low frequencies than when applying higher frequencies. The addition of Sr into simple HA samples resulted in a decrease in the *ε*_2_ values with an increase in the applied frequency (Figure 25b) [101]. The elevated *ε*_2_ values observed at low applied frequencies could be related to dipole oscillations. In contrast, at higher applied frequencies, ionic polarization stops, and ions no longer require energy to rotate their dipoles, leading to a decrease in the *ε*_2_ values [198].

##### Dielectric Loss Tangent

Swain et al. [219] demonstrated that the *tan*(*δ*) values decreased as the sintering temperature increased (Figure 22). This reduction was correlated with the density and grain size of the sintered HA samples [249]. It was observed that *tan*(*δ*) values corresponding to HA samples sintered at both 1250 and 1300 °C were approximately equal. However, for the HA samples sintered at 1200 °C, a higher *tan*(*δ*) was obtained, possibly due to the increased porosity in this specific case [32].

The rise in *tan*(*δ*) values with increasing temperatures observed in all sintered HA samples can be attributed to the generation of defects or free charge carriers. At elevated temperatures, the enhanced mobility of these free charge carriers or defects increases, which leads to the higher *tan*(*δ*) values in the HA system [219,268].

The changes in *tan*(*δ*) values at RT across various applied frequencies ranging from 100 Hz to 1 MHz for all HA/BZT–BCT composites is illustrated in Figure 23 [59]. Notably, for all the investigated composites, lower *tan*(*δ*) values in the frequency range of 1 kHz to 1 MHz were observed [59]. Moreover, the fluctuation of *tan*(*δ*) values at various frequencies concerning the temperature of HA/BZT–BCT composite samples was also investigated (Figure 24). The rise in *tan*(*δ*) values with temperature were linked to the presence of free charge carriers or defects. At elevated temperatures, the enhanced mobility of free charge carriers and defects lead to higher *tan*(*δ*) values in the HA/BZT–BCT composite samples [59].

It should be noted here that the *tan*(*δ*) values exhibited an inverse relationship with the applied frequency [180]. At low applied frequencies, the enhanced *tan*(*δ*) values were attributed to the hopping mechanism of free charge carriers [181]. Meanwhile, at higher frequencies, the dielectric loss was associated with the harmonic oscillator [28]. Mahanty A. and Shikha D. have shown that both intrinsic and extrinsic factors contribute to the change in dielectric loss [177]. The increase in Zn^2+^ concentration in the HA matrix led to a decrease in dielectric loss due to the charge transfer (hopping mechanism) between Ca^2+^ and Zn^2+^ cations. Among all the investigated samples, the Zn-doped (1 wt.%) HA samples exhibited the highest values of dielectric loss, followed by undoped HA and Zn-doped (4 wt.%) HA. It is important to mention that the increment in Zn^2+^ content in HA resulted in larger grain sizes, accompanied by a reduction in resistivity and *ε*_2_ values.

The comportment of *tan*(*δ*) at RT, investigated at frequencies ranging from 10 Hz to 10 MHz, for zero DC bias voltage, is shown in Figure 25c. As one can observe, at low applied frequencies, the investigated samples demonstrated more energy loss than in the case of higher frequencies. With the increase in the applied frequencies, the values of the energy loss decreased. This pattern can be explained as follows: the dipole moment of the (OH^−^) group aligns with the external AC electric field, requiring more energy at low applied frequencies. Consequently, this leads to higher *tan*(*δ*) values. Although, as the frequency increases and reaches higher values, the dipole moment can no longer keep pace with the external AC electric field. This signifies no additional energy requirement, and thus, the *tan*(*δ*) values decrease. At 10 Hz, the *tan*(*δ*) values of the NPs vary, reflecting differences in *ε*_1_ values. Undoped HA samples exhibited specific *tan*(*δ*) values due to polarization. In Sr-doped HA samples, the polarization was shown to decrease due to Sr addition, leading to a reduction in *tan*(*δ*) values. Conversely, in the case of Sr- and Ni-doped HA samples, polarization and, consequently, *tan*(*δ*) values were demonstrated to increase with an increase in the doping concentration. Ideal dielectric materials should possess a high dielectric constant and a low *tan*(*δ*). The stable dielectric constant values across a certain frequency range, combined with low *tan*(*δ*) values, advanced the doped samples as promising candidates for applications in high-frequency devices and the treatment of bone-related issues [101].

### 5.7. Simple, Doped, and Blended Hydroxyapatite Thin Films

Table 3 introduces information related to the materials used, synthesis techniques, and tackled electrical parameters in the case of HA thin films. For more detail on the studies, refer to the corresponding references provided in the last column of Table 3.

#### 5.7.1. Conductivity

**Figure 28:**
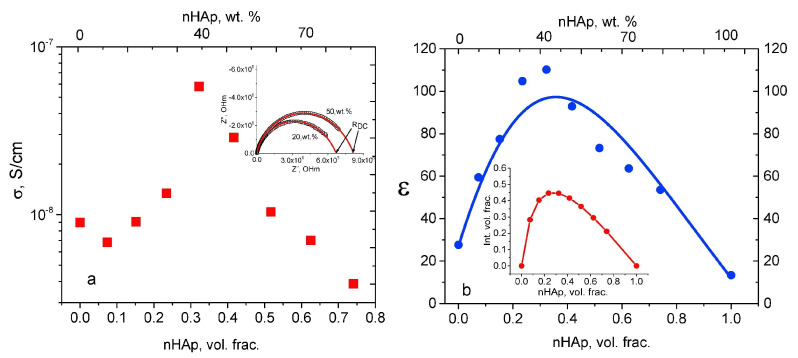
(**a**) Dependence of DC conductivity (*σ_dc_*) of chitosan–nanohydroxyapatite films and (**b**) dependence of dielectric constant in the limit of zero frequency (*ε*—referred to as *ε_S_* in the text) obtained in chitosan–nanohydroxyapatite films with different nanohydroxyapatite concentration points (continuous line shows results of fitting). Reproduced with permission from [164].

The relationship between *σ_dc_* (Figure 28a) and *ε*_1_ (Figure 28b) at the zero-frequency limit (*ε_S_*), concerning HA wt.% and volume fraction, was evidenced in the work of Sanchez et al. [164]. In all cases, there was a peak observed in the dependencies at a concentration of approximatively 30–40 wt.% of nHA (Figure 28). The inset on Figure 28a shows the impedance spectra of chitosan–nHA films obtained at 20 °C for nHA concentrations indicated on the graph. The inset on Figure 28b demonstrates the inferred dependence of the interfacial volume fraction function of the nHA volume fraction and K obtained from fitting.

The *σ_dc_* values demonstrated a significant increase as the nHA concentration increased from 0 to 30–40 wt.% (with a subsequent decrease at higher concentration levels). Such rapid enhancement in property values was associated with the formation of interconnected or percolated microstructures in nanocomposites. The results of this graph demonstrate that, beyond an nHA concentration exceeding 30–40 wt.% (where the dielectric percolation threshold is evident), the interfacial volume fraction decreases as a result of cluster overlap [164]. Moreover, the high ε values obtained in this study compared with other HA–BaTiO_3_ composites should be emphasized: 67.1 vs. 26 (35 vol.% of BaTiO_3_, 10 kHz) [270], 99 vs. 9 (30 wt.% BaTiO_3_, 100 Hz) [271], and 59.4 vs. 12 (10–12 vol.% BaTiO_3_, 1 Hz) [272].

**Figure 29:**
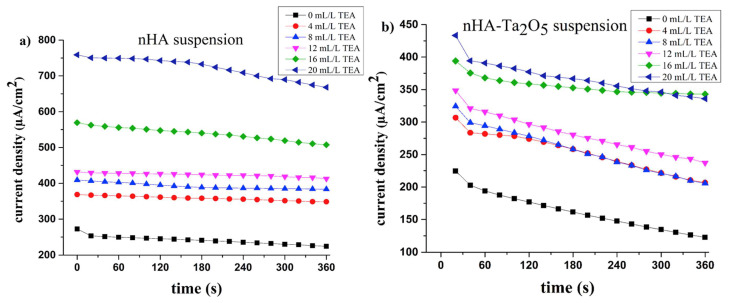
Current density during electrophoretic deposition at 60 V for 360 s from (**a**) nanohydroxyapatite and (**b**) nanohydroxyapatite–Ta_2_O_5_ suspensions with different contents of triethanolamine (TEA). Reproduced with permission from [125].

In their study, Horandghadim N. and Khalil-Allafi J. demonstrated that the inclusion of higher contents of triethanolamine (TEA) in both nHA and nHA–Ta_2_O_5_ suspensions increased the values of the current density (Figure 29) [125].

Therefore, it was found that, at an equivalent value of the TEA, nHA suspensions exhibited higher values of the current densities as compared to nHA–Ta_2_O_5_ suspensions. This result was attributed to their superior electrical conductivity. Another aspect to consider was the decrease in current densities with increasing electrophoretic deposition (EPD) time. This finding was related to the development of insulating ceramic layers formed by nHA and nHA–Ta_2_O_5_ films. These deposits contributed to a voltage drop during constant voltage EPD, which resulted in a subsequent reduction in current density. Moreover, a notable difference in film thickness was observed when performing a comparison between nHA and nHA–Ta_2_O_5_ films (thickness of ~5 µm, obtained at 60 V and 60 s). This reduced thickness was due to the lower electrical conductivity of the nHA–Ta_2_O_5_ suspension compared with the nHA alone [125].

#### 5.7.2. Dielectric Measurements

Further insights into the structure and properties of chitosan (CS)-doped nHA nanocomposite films were obtained through dielectric spectroscopy measurements spanning the frequency range of 40 Hz to 100 MHz. These films displayed distinctive depressed semicircles in their dielectric spectra. By fitting these semicircles using the Cole–Cole expression and the ZView program, values for the DC resistance and capacitance of the films were determined [164]. Additional dielectric spectroscopy measurements in the GHz frequency range were conducted to explore the molecular interactions between the CS matrix and nHA. Previous studies have indicated that, in the frequency range of 10^7^ to 3 × 10^9^ Hz, the dielectric loss dependent on frequency displayed two relaxation processes linked to the rotation of OH^−^ groups (centered around 5 × 10^8^ Hz) and NH_3_ groups of CS (centered around 1 × 10^9^ Hz) [273]. These relaxation processes were also observed in CS-doped nHA films with nHA concentrations below 30 wt.%. However, in nanocomposite films with HA concentrations exceeding 40 wt.%, the relaxation process associated with the rotation of NH_3_ groups disappeared, whereas the amplitude and position related to the rotation of OH^−^ groups remained basically unchanged [164].

#### Dielectric Constant

Sanchez et al. [129] reported that the *ε*_1_ values experienced a significant rise as the nHA concentration increased from 0 to 30–40 wt.%. This sharp increase in property values could be attributed to the formation of connected or percolated microstructures within the nanocomposites [129]. The relationship between *ε*_1_ and nHA concentration resembled the pattern observed for previous investigations, with a peak occurring at a concentration of ~30–40 wt.% nHA [129]. The elevated *ε*_1_ values observed in CS-doped nHA composites are attributed to the robust interfacial *ε*_1_ resulting from the strong interaction between CS an nHA. This enhanced performance of CS-doped nHA compared to cellulose was likely due to the presence of primary amine groups in CS [164].

**Figure 30:**
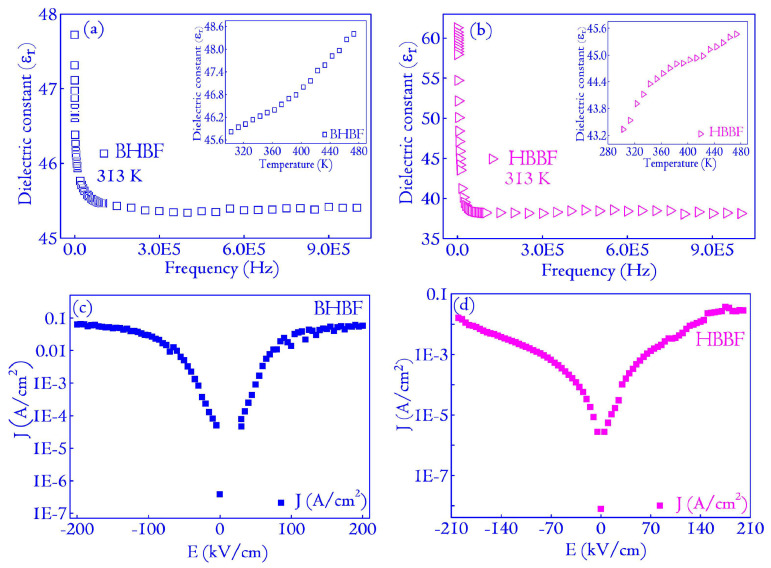
The variation of *ε_r_* (referred to as *ε*_1_ in the text) with respect to frequency and temperature (see inset) for (**a**) bi-layered films of hydroxyapatite onto strontium titanate (BHBF) and (**b**) bi-layered films of strontium titanate onto hydroxyapatite (HBBF) films and (**c**,**d**) leakage current variations. Reproduced with permission from [178].

An analysis using field-emission scanning electron microscopy (FESEM) indicated that the bi-layered films of HA onto strontium titanate (BHBF) exhibited a denser microstructure than the bi-layered films of strontium titanate onto HA (HBBF), where open spaces could be observed between the larger polyhedral grains. This difference in compactness of the microstructure generated variations in density and resulted in subtle differences in the *ε*_1_ values of the bi-layered thin films [274]. The frequency dependence of *ε*_1_ displayed a significant dispersion in bi-layered films, which is a common phenomenon observed in dielectrics [275]. Figure 30a,b illustrates the variation of dielectric properties with frequency, accompanied by changes in temperature, while Figure 30c,d shows the results of the leakage current measurements. It can also be seen that the inferred *ε*_1_ values of BHBF and HBBF films are 45 and 38, respectively, at 1 MHz.

As the frequency raised, changes in the *ε*_1_ values were observed. This phenomenon was explained by the dipoles within the thin films being unable to keep up with the frequency changes of the applied field [68]. Consequently, the thin films’ polarization diminished at higher applied frequencies due to this inability of the dipoles to adjust to the changing frequency of the applied field [178]. Moreover, the increase in the *ε*_1_ values with temperature could be connected to thermal energy, facilitating the rotation of dipoles in the direction of the applied field [64]. In the realm of biomedical applications, the *ε*_1_ values corresponding to coatings on implants could be a crucial parameter in determining the rate of fracture recovery. Medical practices often utilize external electric fields to accelerate fracture healing [276,277,278]. Although the precise mechanism behind this process is not fully understood, its positive effects are well-recognized [279]. In intricate situations requiring implants to facilitate the healing process, the use of an electroactive coating with active polarization capabilities can enhance the recovery process. When subjected to an electric field at specific time intervals, the polarized dielectric layer (consisting of bi-layers of strontium titanate and HA) has the capacity to generate its internal field [280,281]. Additionally, the higher *ε*_1_ values for the BHBF-fabricated structures corresponded to enhanced cell proliferation. Consequently, the healing process accelerated, underscoring the crucial role of *ε*_1_ (linked to polarization) in diverse biological applications [281].

Das et al. [176] documented in their work the changes in the *ε*_1_ values with changes in temperature and applied frequency. The initial dispersion observed in the *ε*_1_ values within the examined frequency range was ascribed to the activation of different polarization mechanisms. The frequency-dependent *ε*_1_ dispersion was explained by Koop’s phenomenological theory, which attributes the initial exponentially high value of *ε*_1_ to the accumulation of space charges at the thin film–electrode interface [282]. With the increase in applied frequency, the polarization mechanisms that can readily track the applied field at low frequencies gradually lag behind, resulting in a decrease in the *ε*_1_ values [176]. Apart from the applied frequencies, the dielectric response of a bio-ceramic coating is influenced by various factors such as grain size, film orientation along specific (hkl) directions, the presence of secondary phases, and/or mechanical stress [283]. It is important to mention that an increase in the *ε*_1_ values with the grain size was also indicated [176].

In the case of thin films, the region around grain boundaries contains defects that introduce states of defect in the bandgap region and facilitate the hopping conduction process [284]. At low applied frequencies, mobile charges quickly accumulate near the interface between grain boundaries. Because of the low applied frequencies, the instantaneous field keeps them accumulated for a longer period compared to higher frequencies. After this duration, the charges reverse direction to the other end of the grain boundary under the influence of the alternating field. This behavior is akin to a small capacitor with a thickness equivalent to the one of the grain boundary. Because the thickness of the grain boundary has a minimal value (as evidenced by the FESEM analysis), the capacitance of the grain boundary is very high. These contributions from the grain boundary also determine an increase in the *ε*_1_ values of the thin films at low applied frequencies [284]. At higher frequencies, the instantaneous fields only act for a brief period before reversing direction, which leads to a rapid reduction in the accumulation of mobile charges at the grain–grain boundary interface. As a consequence, there is a decreased contribution of the grain boundaries to the *ε*_1_ values [284].

The enhanced thin films’ crystallinity is directly associated with their improved dielectric properties. This relationship is evident in the increased *ε*_1_ values with the growing thickness of the thin films, indicating a positive correlation [283]. Furthermore, as the thickness increased, a greater number of dipoles became available to respond to the applied field, thereby making a positive contribution to the *ε*_1_ values. An increase in the *ε*_1_ values with rising temperature was also evidenced. These fluctuations resulted from the thermally activated rotational motion of the dipoles aligned along the field direction [33]. The introduction of temperature provides the necessary rotational energy, enabling the alignment of dipoles along the field direction. This results in an increase in polarization. This phenomenon is particularly noticeable at higher temperatures, as the dipoles, which remain frozen at lower temperatures, become more active due to the additional thermal energy [275].

##### Tangent Loss

Mitra et al. [269] represented the *tan*(*δ*) values as a function of the logarithm of frequency. For PMMA–HA, the *tan*(*δ*) values peaked at a maximum of 23.90°, when the frequency was at a logarithmic value of ~131 Hz. Subsequently, it steadily decreased as the logarithmic frequency value increased until it stabilized within the range of 10^5^–10^6^ Hz. The elevated values of *tan*(*δ*) at low applied frequencies could be attributed to the effectiveness of grain boundaries, which surpass individual grains in influencing the dielectric behavior [285]. In the case of PMMA–HA/Al_2_O_3_, *tan*(*δ*) was shown to reach a constant value of 0.098°, remaining parallel to the logarithmic frequency value within the specified range. The consistent *tan*(*δ*) values observed in the coated samples could be explained by the nonconductive nature of α-Al_2_O_3_ and the relatively smaller grain boundaries compared to PMMA–HA [269]. This stabilization in *tan*(*δ*) caused by increasing the applied frequency is due to the characteristics of the doped PMMA, which reduces the grain size, contributing to the overall decrease in *tan*(*δ*) values.

## 6. Conclusions and Future Perspectives

In recent years, there have been some interesting studies conducted that have renewed attention on the electrical and dielectric properties of hydroxyapatite (HA). This resurgence has been marked by significant advancements in piezoelectric HA and the creation of electrostatic domains, with potential physiological implications.

The aim of this review was to highlight the importance of investigating the electrical and dielectric characteristics of HA-based biomaterials, ranging from powders and pellets to thin films, with a particular focus on the impact of various dopants used. It has been shown that each dopant possesses unique and superior properties (to pristine HA), capable of enhancing the overall characteristics of the produced structures. In the case of powders/nanoparticles, the inferred *ε*_1_ and *σ_ac_* values were evidenced to be influenced by the doping agents. Thus, at increased dopant concentrations, *σ_ac_* evidenced maximum values, while the *ε*_1_ values were shown to be consistently higher than those corresponding to undoped HA. The dielectric loss (*ε*_2_) values corresponding to doped HA samples were demonstrated to be inferior to those corresponding to undoped HA. Moreover, the samples containing the highest doping concentration were shown to exhibit progressive increases in the real permittivity values, as compared to pristine HA samples. In the case of pellets, the values of total HA resistance were shown to decrease with the doping concentrations. Compared with pristine HA, the doped HA samples displayed higher ionic conductivity in the low-frequency region. It was also demonstrated that the permittivity, *σ_ac_*, and *ε*_1_ values corresponding to doped HA samples were superior to the ones corresponding to undoped HA. The same observation as in the case of powders/nanoparticles was made in the *ε*_2_ values, which diminished as the doping concentration increased; this observation is in contrast to that of undoped HA powders and nanoparticles. The addition of dopants to the HA structure and an increase in the applied frequency resulted in larger grain sizes, which was accompanied by a reduction in resistivity and *ε*_2_ values. In the case of thin films, the *ε*_1_ values experienced a significant rise as the HA concentration increased.

Nevertheless, comprehensive investigations are still necessary for the fine tuning of the dopant concentrations within the HA structure, focusing on its physical, mechanical, antibacterial, antifungal, electrical, and dielectric properties. Moreover, innovative solutions containing superior doping agents or blends (i.e., conductive polymers) could provide new avenues for the development of the next generation of high-performance HA-based materials for top orthopedic and dental implant applications.

## Figures and Tables

**Table 1:** Information related to the used materials, techniques, and tackled electrical parameters in the case of simple and doped hydroxyapatite powders/nanoparticles.

Material	Dopant Concentration	Synthesis Method	Investigated Electrical/Dielectric Parameters	Frequency Range	Temperature [K]	Refs.
Te-doped HA nanorods/nanosheets	0.04; 0.08; 0.16; 0.24 wt.%	Microwave-assisted technique	Dielectric constant, dielectric loss, AC conductivity	3 kHz–10 MHz	RT	[182]
Sr-doped HA nanorods	0.03; 0.06; 0.12; 0.24; 0.48	Microwave/hydrothermal technique	Dielectric constant, dielectric loss, AC conductivity	3 kHz–10 MHz	RT	[97]
Li-doped HA nanocomposites	0; 1; 5; 10; 20; 30; 40 wt.%	Sol-gel technique	Dielectric, AC conductivity	3 kHz–10 MHz	RT	[7]
Cu-doped HA	0.2; 0.4; 0.6; 0.8 mol	Co-precipitation technique	Impedance, tangent loss, dielectric constant	–	–	[183]
Ag-doped nano-Sr apatite particles	2; 5; 8; 11 mol	Hydrothermal technique	Dielectric constant, dielectric loss, AC conductivity	1 kHz–2 MHz	RT	[175]
V-doped HA nanorods	1; 5; 10; 20; 30%	Sol-gel/ hydrothermal technique	Dielectric constant, AC conductivity	3 kHz–10 MHz	RT	[105]
(La, Ba, Fe, and Zn)-doped HA	0.02; 0.05%	Sol-gel technique	AC conductivity, impedance	50 Hz–5 MHz	309–688	[26]
Ag-doped HA	0; 0.2; 0.4; 0.8; 1 mol	In situ hydrothermal technique	Dielectric permittivity, ionic conductivity, complex impedance	–	613	[85]
Fe/Cu-co-doped HA	(0.29 at.%)/0; 0.29; 0.58; 0.87	Wet-chemical technique	Dielectric constant, dielectric loss, AC conductivity	10 Hz–10 MHz	RT	[170]
HA/hardystonite/copper oxide	0; 2.5; 5; 7.5 wt.%	Mechanochemical synthesis technique	AC conductivity, dielectric constant, dielectric loss	1–20 MHz	RT	[115]
Ag-doped HA NPs	10; 20; 30; 40 wt.%	Casting technique	Dielectric constant, AC conductivity	100 kHz–1 MHz	303–405	[154]
HA NPs	–	Autoclave assisted hydrothermal technique	Dielectric constant, dielectric loss, AC conductivity	1 kHz–5 MHz	RT	[184]
Sr/Ni co-doped HA	0; 0.37; 0.74; 1.11 at.%	Wet chemical technique	Dielectric constant, dielectric loss	1 kHz–2 MHz	293–473	[101]

**Table 2:** Information related to the materials used, techniques, and tackled electrical parameters in the case of simple and doped hydroxyapatite pellets.

Material	Dopant Concentration	Synthesis Method	Investigated Electrical/Dielectric Parameters	Frequency Range	Temperature [K]	Ref.
Ce-doped HA	1; 1.5; 1.7%	Wet precipitation technique	Dielectric constant, dielectric loss, AC conductivity	1–10 MHz	RT	[218]
Ga-doped HA	–	Precipitation technique	Dielectric constant, dielectric loss, AC conductivity	100 Hz–1 MHz	RT	[171]
Cr-doped HA	–	Co-precipitation technique	Dielectric constant, dielectric loss, AC conductivity, permittivity	1 Hz–10 MHz	RT	[169]
HA ceramics	–	Mechanochemical technique	Dielectric constant, dielectric loss	1 kHz–1 MHz	RT	[219]
xHA/(1-x)BZT-BCT	5; 10; 15; 20 wt.%	High energy ball milling assisted solid-state reaction route	Dielectric constant, dielectric loss	100 Hz–1 MHz	RT	[59]
BaTiO_3_-doped HA	100/0; 90/10; 70/30; 50/50; 30/70; 10/90; 0/100	High-speed mechanical stirring technique	Dielectric constant	100 Hz–100 kHz	RT	[220]
Zn-doped HA	1; 2; 3; 4 wt.%	Sol-gel technique	Dielectric constant, dielectric loss, Impedance	–	–	[177]
Mg- and Ag-doped HA	1; 2; 3; 4 wt.%	Sol-gel wet chemical technique	Dielectric loss, Impedance	100 Hz–10 MHz	–	[179]

**Table 3:** Information related to the materials used, techniques, and tackled electrical parameters in the case of simple and doped hydroxyapatite thin films.

Material	Dopant/Blend Concentration	Synthesis Method	Investigated Electrical/Dielectric Parameters	Frequency Range	Temperature [K]	Ref.
Chitosan–HA	0; 10; 20; 30; 40; 50; 60; 70; 80 wt.%	Solvent cast technique	Dielectric constant, dielectric loss, conductivity	40 Hz–110 MHz	293–473	[164]
T_2_O_5_–HA	20 wt.%	Electrophoretic deposition	Conductivity	–	–	[125]
BaSrTiO_3_–HA		Radio frequency magnetron sputtering	Dielectric constant	1Hz–1 MHz	–	[178]
SrTiO_3_–HA	80 at.%	Radio frequency magnetron sputtering	Dielectric constant	–	–	[176]
PMMA–HA	–	Sol gel technique	Dielectric loss, impedance	–	–	[269]

## Data Availability

Not applicable.
